# Clonal Dynamics Reveal Two Distinct Populations of Basal Cells in Slow-Turnover Airway Epithelium

**DOI:** 10.1016/j.celrep.2015.06.011

**Published:** 2015-06-25

**Authors:** Julie K. Watson, Steffen Rulands, Adam C. Wilkinson, Aline Wuidart, Marielle Ousset, Alexandra Van Keymeulen, Berthold Göttgens, Cédric Blanpain, Benjamin D. Simons, Emma L. Rawlins

**Affiliations:** 1 Wellcome Trust/CRUK Gurdon Institute, University of Cambridge, Cambridge CB2 1QN, UK; 2 Wellcome Trust-MRC Stem Cell Institute University of Cambridge, Cambridge CB2 3EG, UK; 3 Department of Pathology, University of Cambridge, Cambridge CB2 3EG, UK; 4 Cavendish Laboratory, Department of Physics, J. J. Thomson Avenue, Cambridge CB3 0HE, UK; 5 Department of Haematology, University of Cambridge, Hills Road, Cambridge CB2 0XY, UK; 6 Cambridge Institute for Medical Research, University of Cambridge, Hills Road, Cambridge CB2 0XY, UK; 7 Institut de Recherche Interdisciplinaire en Biologie Humaine et Moléculaire, Université Libre de Bruxelles, Brussels 1070, Belgium; 8 Welbio, Université Libre de Bruxelles, Brussels 1070, Belgium

## Abstract

Epithelial lineages have been studied at cellular resolution in multiple organs that turn over rapidly. However, many epithelia, including those of the lung, liver, pancreas, and prostate, turn over slowly and may be regulated differently. We investigated the mouse tracheal epithelial lineage at homeostasis by using long-term clonal analysis and mathematical modeling. This pseudostratified epithelium contains basal cells and secretory and multiciliated luminal cells. Our analysis revealed that basal cells are heterogeneous, comprising approximately equal numbers of multipotent stem cells and committed precursors, which persist in the basal layer for 11 days before differentiating to luminal fate. We confirmed the molecular and functional differences within the basal population by using single-cell qRT-PCR and further lineage labeling. Additionally, we show that self-renewal of short-lived secretory cells is a feature of homeostasis. We have thus revealed early luminal commitment of cells that are morphologically indistinguishable from stem cells.

## Highlights

• Clonal analysis for determining homeostatic tracheal epithelial cell hierarchy
• Basal cells comprise two subpopulations: stem cells and luminal precursors
• Luminal secretory cells are short-lived and self-renewing
• Secretory cells are the major steady-state source of new ciliated cells

## Graphical Abstract

**Figure:**
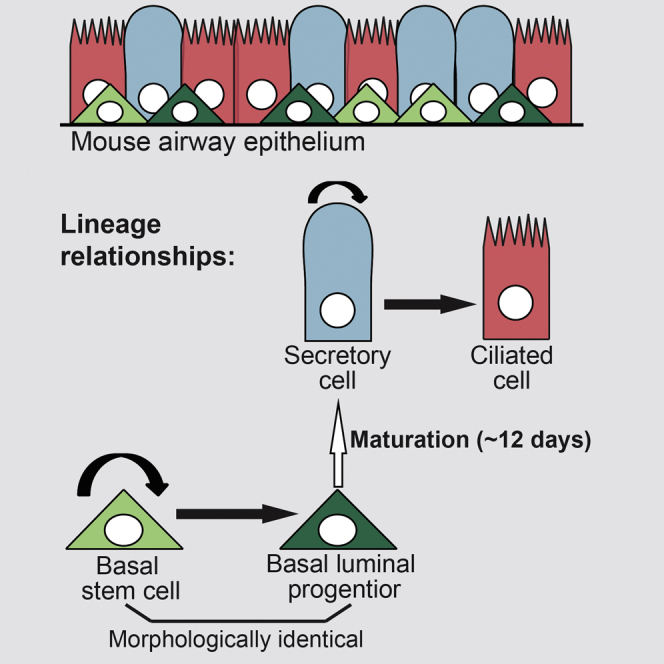

## Teaser

Using clonal analysis, mathematical modeling, and single-cell qRT-PCR, Watson et al. define the homeostatic tracheal epithelial lineage. The epithelium contains two major, equally distributed subpopulations of basal cells: stem cells and long-lived precursors that are already committed to differentiation.

## Introduction

The mouse trachea contains three major cell types: TRP63^+^, KRT5^+^ basal cells (BCs); luminal secretory cells (SecCs, mostly Scgb1a1^+^ Club/Clara-like cells); and luminal ciliated cells (CCs) (Rock et al., 2010). Previous population-level lineage tracing using transgenic *Tg*(*KRT5-CreER)* mice demonstrated that BCs include self-renewing stem cells involved in tracheal growth, homeostasis (at least for up to 16 weeks), and repair (Rock et al., 2009). However, it is not known if BCs are a functionally heterogeneous population. A subset of tracheal BCs (<20%) expressing *Krt14* (*Keratin 14*) was suggested to be a unipotent self-renewing subpopulation at homeostasis (Ghosh et al., 2011). Similar unipotent BCs have been postulated following injury and in xenografts (Engelhardt et al., 1995; Ghosh et al., 2011; Hong et al., 2004). Other repair studies described an early progenitor (EP) cell as a proliferative KRT8^+^ (luminal type cytokeratin), TRP63^−^ cell derived from BCs and controlled by Notch signaling (Paul et al., 2014; Rock et al., 2011). In development, KRT5^+^ TRP63^−^ cells with basal morphology have recently been described in germline *Notch3* mutants and in embryonic lungs deleted for *Ezh2* (Mori et al., 2015; Snitow et al., 2015), leading to the speculation that these are precursors of luminal cells. Subsequently, an independent study showed that a population of adult BCs (∼12% of steady-state total), which express low levels of transcription factors usually found in more differentiated cells, are able to contribute disproportionally to regeneration following injury (Pardo-Saganta et al., 2015). However, none of these studies investigated the adult airway lineage at steady state, leaving key questions unanswered. In particular, is there is an engrained proliferative heterogeneity in the steady-state basal layer? If so, what is the lineage relationship of cells within the basal layer, and how do they connect to the luminal compartments? How do distinct subpopulations of BCs function to maintain normal homeostasis?

Within luminal cells, population lineage-labeling studies had shown that SecCs can self-renew and generate CCs, but their relative contribution to homeostasis was unclear (Rawlins et al., 2009). CCs are post-mitotic, with an average loss-rate of ∼6 months in the trachea (Rawlins and Hogan, 2008; Rawlins et al., 2007). Molecular signals controlling the tracheal epithelium are being determined (Brechbuhl et al., 2011; Giangreco et al., 2012; Lu et al., 2013; Paul et al., 2014; Rock et al., 2011; Zhao et al., 2014). However, the lack of a clearly defined epithelial lineage impedes analysis of molecular function at cellular resolution. Human airways have a very similar cell lineage to mouse trachea (Engelhardt et al., 1995; Hackett et al., 2011; Hajj et al., 2007; Teixeira et al., 2013), but the limited resolution for lineage studies in human means that complementary mouse analysis is required to determine the detailed cellular hierarchy. Here, we use clonal lineage labeling, coupled with biophysical modeling and single-cell molecular analysis, to determine the heterogeneity and functions of BCs and SecCs in the homeostatic mouse tracheal epithelium. We have rigorously obtained quantitative measures of division rates, cell-type abundance, and rates of differentiation/loss. The model that we present thus provides a new experimental and theoretical foundation for studies of airway homeostasis, injury, and disease. Moreover, we reveal an unexpected mechanism of epithelial maintenance in a slowly proliferating tissue: widespread early luminal commitment in cells that are morphologically indistinguishable from stem cells.

## Results

### Clonal-Level Lineage Analysis of BCs in the Steady-State Tracheal Epithelium Suggests a Proliferative Hierarchy and the Presence of More Than One BC Subpopulation

**Figure 1:**
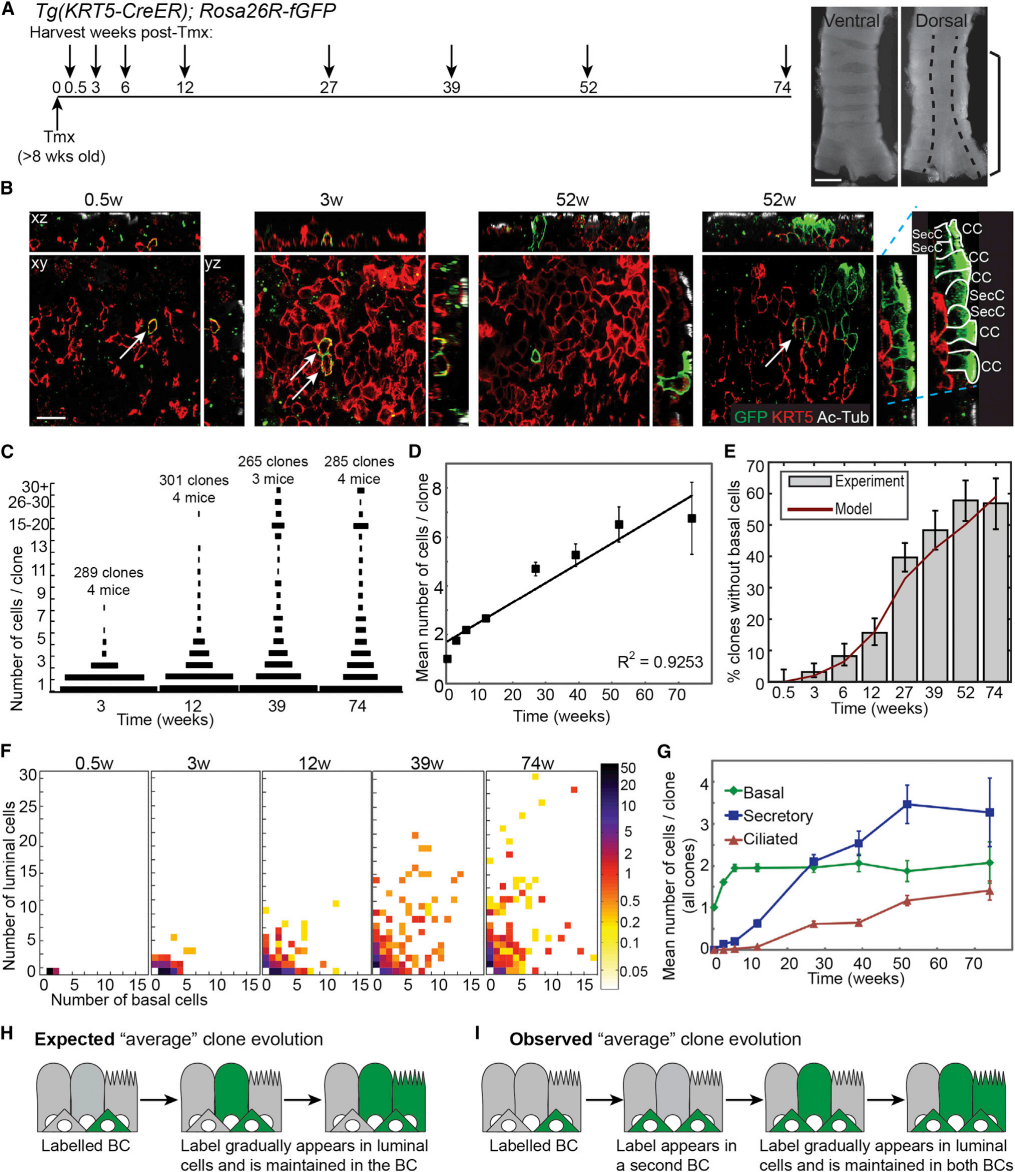
Clonal-Level Lineage Analysis of Basal Cells in the Steady-State Tracheal Epithelium (A) Schematic of the *Tg(KRT5-CreER); Rosa26R-fGFP* lineage-labeling experiment. Ventral and dorsal trachea images illustrate approximate region analyzed in all experiments (brackets on dorsal side) and location of dorsal longitudinal smooth muscle (dashed line). Scale bar, 1 mm. (B) Representative single confocal xy planes of the basal area of the epithelium with corresponding xz and yz reconstructions at 0.5, 3, and 52 weeks post-induction. Green, lineage label; red, KRT5 (BCs); white, acetylated tubulin (cilia). BCs were scored as KRT5^+^, and ciliated cells as KRT5^−^, ac-Tub^+^. Secretory cells were scored as KRT5^−^, ac-TUB^−^ cells whose apical surface reaches the tracheal lumen (this definition will also include a small number of other cell types such as brush and neuroendocrine cells). See right yz panel for examples of labeled luminal cells. GFP-labeled BCs are indicated by arrows on xy views. Scale bar, 15 μm. (C) Size distributions of clones at 3, 12, 39, and 74 weeks post-induction. Length of bar represents frequency. (D) Plot of the mean number of total cells per clone (all clones included). Error bars represent SEM. (E) Plot of the percentage of clones that do not contain any labeled BCs (gray bars). Error bars represent 95% confidence intervals. Red line represents values predicted by the BC model (see Figures 2A–2C). (F) Heatmaps to show the distribution of basal and luminal cells within each clone at 0.5, 3, 12, 39, and 74 weeks. Colors represent percentage frequency of each clone type on a log scale. (G) Plot of the mean number of cells of each type per clone (all clones included). Green, BCs; blue, secretory cells; red, ciliated cells. Error bars represent SEM. (H and I) Analysis of our lineage-labeling data at a population level (see Figures 1C–1G) is inconsistent with an homogenous BC population (depicted in H) and suggests there are two BC subtypes and, moreover, that our experiments labeled a basal stem cell (BSC) that maintains both the luminal cells and the second BC population (depicted in I). See also Figures S1 and S2.

To study maintenance of the tracheal epithelium, we first tested whether homeostasis was maintained during our time course by analyzing cell proliferation, composition, density, and tracheal size (Figure S1). This confirmed that the tissue was homeostatic for most of the time course, although the proportion of CCs increased by ∼30%, and cell density decreased by ∼30%, in older animals (1 year post-labeling) consistent with previous data (Wansleeben et al., 2014). To label individual *Krt5*^*+*^ BCs, we used a transgenic mouse line, *Tg(KRT5-CreER)* (Rock et al., 2009), with a *Rosa26*-reporter driving membrane-targeted (farnesylated) EGFP (Rawlins et al., 2009). Exposure of adult (>8 weeks) *Tg(KRT5-CreER);Rosa26R-fGFP* mice to a single low dose of tamoxifen (tmx) resulted in scattered individual lineage-labeled BCs in the distal trachea (from the carina to six cartilage rings above on the dorsal side only; Figure 1A). Negligible labeling was detected in animals without tmx exposure (two clones of one to six cells in two out of four mice at 9–11 months age). Tracheas were harvested at intervals from 0.5 to 74 weeks post-tmx (Figure 1A) and whole-mount immunostained to determine clone size and composition by confocal microscopy (Figure 1B; Table S1). Clonal density varied between mice. However, clones were always more frequently located above the dorsal longitudinal smooth muscle, rather than the cartilage rings. As clones grew, they remained cohesive, suggesting little cell motility at steady state. Moreover, some larger clones were observed to span the muscle-cartilage junction, showing that this is not a compartment boundary.

At 0.5 weeks post-induction, clones consisted predominantly of single BCs (99% one BC: 1% two BCs; n = 102 clones, four mice). Rare labeled SecCs and CCs appeared at 3 and 6 weeks post-induction, respectively, and their numbers rose steadily thereafter (Figures 1B, 1F, and 1G), confirming that BCs generate luminal cells. The size distribution of clones became increasingly heterogeneous, but mean clone size increased in a remarkably linear fashion over time (Figures 1C and 1D), consistent with self-renewal of the labeled cells. From 3 weeks post-induction, clones that contained only luminal (secretory and/or ciliated) cells and no BCs emerged, showing that some labeled BCs are lost to differentiation (Figures 1E and 1F). The increasingly heterogeneous clonal composition and the emergence of clones lacking BCs indicated that BCs can divide symmetrically and asymmetrically at steady state, similar to progenitors in the inter-follicular epidermis (Clayton et al., 2007; Mascré et al., 2012). To determine if any of the clonal heterogeneity could be attributed to animal-specific or regional differences in the trachea, we graphed clones separately based on sex or location (Figure S2). Although we observed some small systematic differences in the behavior of clones located over cartilage rings versus dorsal longitudinal muscle, these were statistically insignificant, and we have treated the distal-dorsal trachea as a single region (Supplemental Theory).

Strikingly, the mean number of BCs per clone rose abruptly from an average of one to approximately two cells by 6 weeks post-induction and thereafter remained remarkably constant over the following 68 weeks (Figure 1G). Moreover, most two-cell clones (88%) at 3 weeks post-induction contained precisely two BCs. This was unexpected for a population in homeostasis. By definition, the overall distribution of cell types within a tissue stays constant at homeostasis. Hence, if the transgene targets all BCs in a representative manner, the mean number of BCs per clone should remain at one. The abrupt increase in the average number of BCs per clone, and its near saturation at approximately two BCs per clone over the long-term, indicate that the lineage-labeling assay preferentially targets a subpopulation of BCs, which maintains a second initially unlabeled population (Figures 1H and 1I). We therefore postulated that *Krt5*^*+*^ BCs contain two discrete populations organized in a hierarchy: a multipotent basal stem cell (BSC; preferentially targeted by the assay) and an additional BC subtype.

### Biophysical Modeling of the Behavior of Homeostatic Tracheal BCs

To resolve the cellular hierarchy, proliferation kinetics, and fate potential in the trachea, we used a biophysical modeling approach. We sought the simplest model that could describe the observed distributions of clone sizes and composition and provide testable predictions. To constrain the number of model parameters, we first used the *Tg(KRT5-CreER); Rosa26R-fGFP* clonal assay to infer the dynamics of the BCs alone. Independently, we employed a second lineage-labeling assay that targets SecCs to infer the dynamics of luminal cells alone. Finally, we used the basal and luminal clonal fate data from the *Tg(KRT5-CreER); Rosa26R-fGFP* experiment to challenge the predictions of the model.

**Figure 2:**
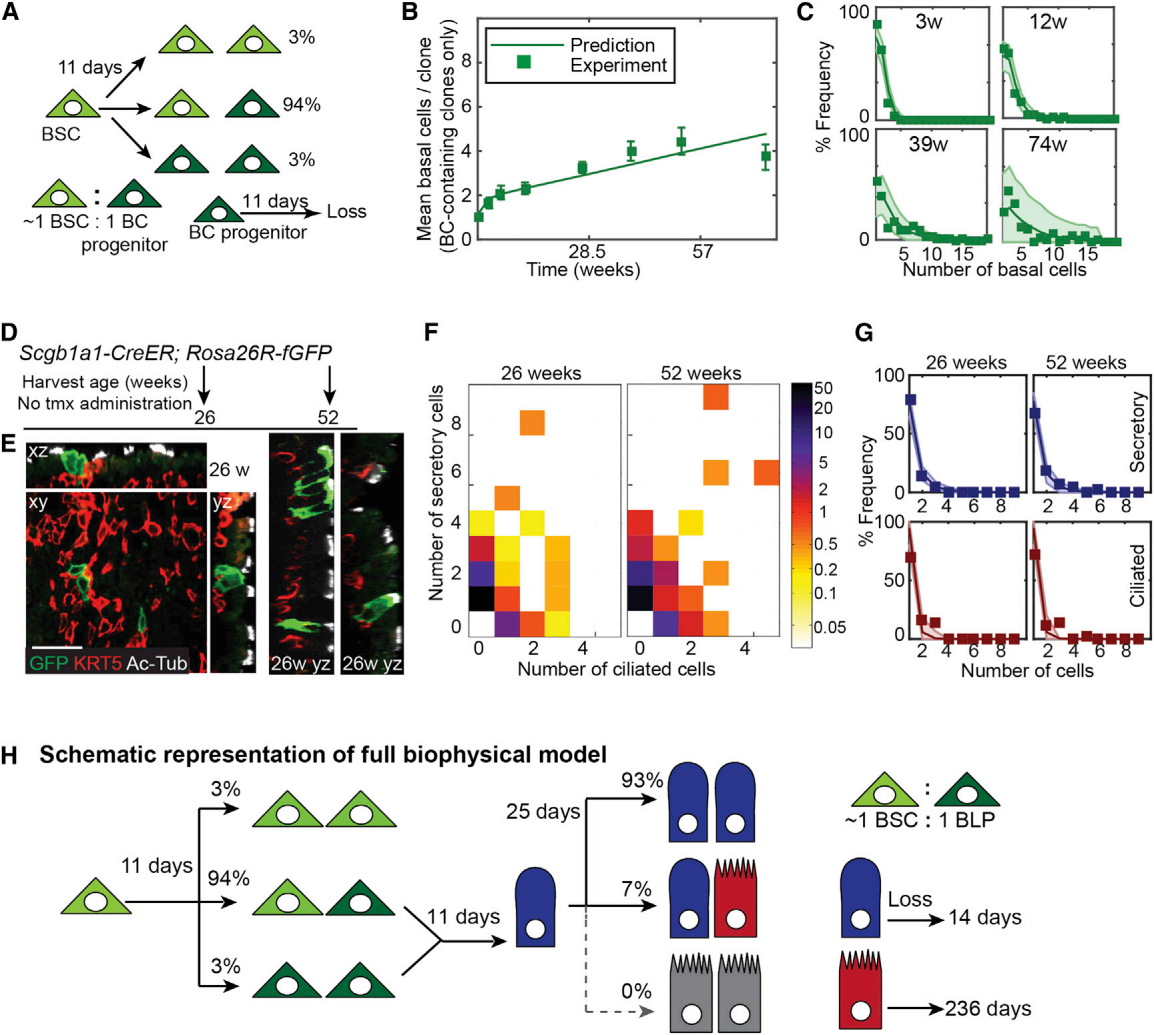
Lineage Model Development for the Basal and Luminal Epithelial Cells (A) Cellular representation of the mathematical model for BC behavior. Basal stem cells (BSCs) and BC progenitors are present in the tissue in an ∼1:1 ratio. BSCs divide symmetrically or asymmetrically every 11 days to either self-renew or produce BC progenitors in a balanced manner. BC progenitors are lost, to either further differentiation or death, at a rate of every 11 days. (B) Plot showing the experimental observation (boxes) and model prediction (line) of mean number of basal cells per clone (only clones containing BCs are included in this plot and used for the generation of the BC model). Error bars represent SEM. (C) Percentage frequency distribution of basal cell numbers per clone at 3, 12, 39, and 74 weeks. Boxes, experimental observations; dark line, model prediction of the BC model; shaded area, 95% confidence intervals of the model. (D) Schematic of *Scgb1a1-CreER; Rosa26R-fGFP* lineage-labeling experiment. (E) Representative xy, yz, and xz confocal sections of three different clones at 26 weeks. (By 26 weeks, the clonal size and composition had already reached a steady state, and these images are representative of the full data range.) Green, lineage label; red, KRT5 (BCs); white, acetylated tubulin (cilia). Scale bar, 25 μm. (F) Heatmaps to show the distribution of secretory and ciliated cells observed in all *Scgb1a1-CreER; Rosa26R-fGFP* clones over time. Colors represent percentage frequency of each clone type on a log scale. (G) Frequency distribution of secretory or ciliated cell numbers per clone at 26 and 52 weeks. Blue, secretory; red, ciliated cells. Boxes, experimental observations; dark line, predictions of luminal cell model; shaded area, 95% confidence intervals of the luminal cell model. (H) Cellular representation of the combined mathematical model. BSCs (light green) divide on average once every 11 days in a balanced manner to produce equal numbers of new BSCs and BLPs (dark green). (Ratio of BSCs: BLPs within the epithelium is ∼1:1.) BLPs mature to a luminal cell fate (depicted as secretory, but could be ciliated) once every 11 days. Secretory cells (blue) divide every 25 days to generate two new secretory cells (93%) or one secretory and 1 ciliated (red) cell. The relative proportions of secretory and ciliated cells in the epithelium (∼2:1) are maintained by differential rates of loss (every 14 days for secretory cells and every 236 days for ciliated cells).

Focusing on BCs alone, we investigated whether a model involving two distinct BC subtypes organized in a hierarchy could predict the complex clonal evolution observed. We proposed a model in which a self-renewing basal population (termed BSCs) can divide asymmetrically, giving rise to a BSC and a BC of a second subtype (termed “basal progenitor”), with the two cell types present in approximately equal numbers within the tissue. To account for clonal loss from the basal compartment, we further conjectured that BSCs are also capable of symmetrical cell division, resulting in two BSCs or two basal progenitors. To maintain homeostasis, these two outcomes must occur with equal probability (Figure 2A). From a fit of this stochastic model to the clonal data, we found that a BSC divides, on average, every 11 ± (confidence interval: 4, 4) days. The vast majority of divisions 94 ± (3, 2)% result in asymmetric fate outcome, with the remainder leading to balanced BSC loss/replacement. Basal progenitors are lost, either to further differentiation or death, on average every 11 ± (7, 4) days. With this simple model, we obtained an excellent fit to the entire range of BC clonal data (Figures 2B and 2C; Supplemental Theory).

### Clonal Lineage Analysis of Luminal Cells Shows that SecCs Are Short-Lived and Preferentially Self-Renew at Homeostasis

To independently investigate luminal cell behavior, we analyzed the trachea of 6- and 12-month-old *Scgb1a1-CreER; Rosa26R-fGFP* mice (SecC labeling) in which there was a low rate of spontaneous recombination of the reporter in SecCs in the absence of tmx (Figures 2D and 2E). We observed scattered clones throughout the epithelium and focused on the distal-dorsal region used in our BC experiments. Strikingly, the vast majority of clones were small (compare Figures 2F and 1F), even though SecCs divide (Figure S1D), suggesting that the loss rate of SecCs exceeds their rate of self-renewal, necessitating constant replacement by the multipotent BSCs. Nevertheless, we observed labeled CCs, confirming that the SecCs do contribute to the CC lineage at steady state (Figures 2E and 2F; Table S1).

To infer the rules of lineage specification in the luminal cells, we again made use of a simple biophysical modeling scheme, whereby SecCs may divide symmetrically or asymmetrically or are lost through turnover, and CCs are post-mitotic and lost at a rate of once every 6 months (Rawlins and Hogan, 2008). Taking into account the fact that labeling occurs continuously and sporadically, we found that SecCs divide, on average, every 25 ± (7, 5) days with almost all divisions (93 ± [2, 2]%) leading to symmetrical duplication. The production of SecCs through differentiation of BCs and self-duplication is balanced by a loss rate of once per 14 ± (2, 2) days. From the quality of the fit to the data (Figure 2G), we can infer that, at any given time, 44% of SecCs are derived from the proliferation of a SecC and 56% from differentiation of BCs (Supplemental Theory). Thus SecCs make an important contribution to tracheal homeostasis but do not function as a traditionally defined transit-amplifying population.

### Combining the Basal and Luminal Lineage Models Accurately Predicts the Range of the Full *KRT5* Clonal Dataset

**Figure 3:**
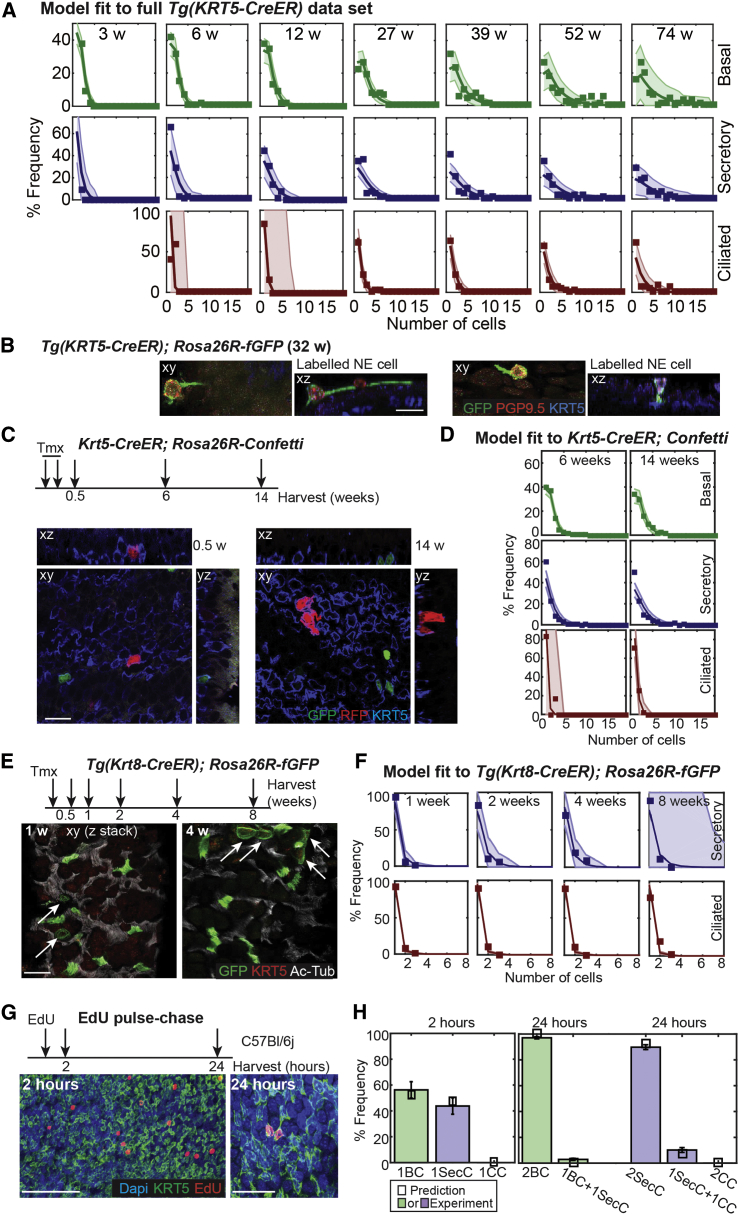
Testing the Tracheal Epithelial Lineage Model (A) Plots showing the fit of the combined (basal and luminal cells) mathematical model to the full *Tg(KRT5-CreER); Rosa26R-fGFP* dataset (all clones included) throughout the time course. Frequency distribution of basal (green), secretory (blue) and ciliated (red) cell numbers per clone. Boxes, experimental observation; dark line, predictions of combined model; shaded area, 95% confidence intervals of the combined model. (B) Representative confocal xy and corresponding xz projections of lineage-labeled NE cells in *Tg(KRT5-CreER); Rosa26R-fGFP* trachea harvested 32 weeks post-induction. Green, GFP (lineage-label); red, PGP9.5 (NE cells); blue, KRT5 (BCs). Scale bar, 10 μm. (C) *Krt5-CreER; Rosa26R-Confetti* mice were labeled and followed for 14 weeks. Representative single confocal xy planes of the basal area of the whole-mount epithelium with the corresponding xz and yz reconstructions at 0.5 and 14 weeks post-induction. Green, nGFP (lineage label); red, RFP (lineage label); blue, KRT5 (BCs). Scale bar, 15 μm. (D) Plots showing the fit of the combined mathematical model to the *Krt5-CreER; Rosa26R-Confetti* dataset at 6 and 14 weeks post-induction. Frequency distribution of basal (green), secretory (blue), and ciliated (red) cell numbers per clone. Boxes, experimental observation; dark line, prediction of combined model; shaded area, 95% confidence intervals of the combined model. (E) Experimental schematic and confocal projections (z stacks) of apical regions from whole-mount lineage-labeled *Tg(Krt8-CreER); Rosa26R-fGFP* tracheal preparations. Green, GFP (lineage label); red, KRT5 (BCs not visible in these projections, but scored in every image); white, acetylated tubulin (cilia). Scale bar, 20 μm. Arrows indicate lineage-labeled SecCs. The other GFP^+^ cells are all ciliated cells. (F) Plots showing the fit of the luminal mathematical model to the *Tg(Krt8-CreER); Rosa26R-fGFP* dataset. Frequency distribution of secretory (blue) and ciliated (red) cell numbers per clone. Boxes, experimental observation; dark line, prediction; shaded area, 95% confidence intervals of the model. (G) Experimental schematic and single confocal planes of the basal region from whole-mount tracheal preparations showing EdU incorporation at 2 and 24 hr post-exposure in wild-type adults. Green, KRT5 (basal cells). Red, EdU (cells in S phase). Blue, DAPI (nuclei). Scale bar represents 100 μm (2 hr) or 25 μm (24 hr). (H) Plot showing the percentage frequency of types of dividing cells observed (colored bars) compared to predictions of the combined model (small black boxes). Error bars represent SEM; n = 3 mice at each time. (Note: only single EdU^+^ cells at 2 hr and pairs of EdU^+^ cells at 24 hr were included in the analysis. The full dataset included some groups of more than two cells and some single cells at 24 hr, likely resulting from clone merging or EdU toxicity, respectively; Table S1.)

With the dynamics of the basal and luminal cells defined separately, we then asked whether a combined model could predict the full range of complex clonal data in the *Tg(KRT5-CreER); Rosa26R-fGFP* mice. The simplest combined model is one in which basal progenitors represent cells committed to a luminal (depicted as secretory) fate (called basal luminal precursors [BLPs]) (Figure 2H). Indeed, if these cells differentiate directly to SecCs without division, the combined model accurately predicts the overall detailed clonal variation throughout the entire long-term time course (Figure 3A). Production of new CCs happens rarely at homeostasis and, within the confidence limits of our model, can be entirely accounted for by division of SecCs. However, the resolving power of our clonal analysis is limited for rare events and the direct steady-state production of CCs from the BLPs cannot be altogether ruled out.

Are the BLPs a transit-amplifying population? To meet the traditional definition of a transit-amplifying cell population (Watt and Hogan, 2000), the BLPs would need to self-renew symmetrically at a greater rate than the stem cell in order to increase the pool of undifferentiated cells. From the clonal data, we cannot exclude the possibility that BLPs can self-renew symmetrically. However, our observations of the rate of bromodeoxyuridine (BrdU) incorporation in the steady-state trachea (Figures S1A–S1D) set the overall rate of all BC divisions to 0.09 per day. If we allow BLPs to divide symmetrically in the model, the other parameters are such that we estimate the maximum rate at which BLP division could occur is 0.045 cell divisions per day (Supplemental Theory). This is half the total number of observed cell divisions in the basal layer, suggesting that the BLPs do not divide at a greater rate than the BSCs and are thus not a traditionally defined transit-amplifying population.

A close inspection of the fit to the *Tg(KRT5-CreER); Rosa26R-fGFP* data reveals that the model provides a consistent slight underestimate of single SecC clones (Figure 3A, blue charts). We hypothesized that the excess single SecCs observed represent rare brush and neuroendocrine (NE) cells that are of unknown origin in the trachea (Krasteva et al., 2011; Saunders et al., 2013). Indeed, when we stained *Tg(KRT5-CreER); Rosa26R-fGFP* tracheas for an NE marker, we identified lineage-traced NE cells (Figure 3B). This demonstrates that tracheal NE cells can be derived from BSCs. However, brush and NE cells were indistinguishable from SecCs in our quantitative experiments, and we have not specifically included them in the model.

### Independent Clonal and Proliferation Analysis Supports the Tracheal Lineage Model

To further challenge the validity of the model, we repeated part of the BC lineage-tracing time course by using an independent *Krt5-CreER* knockin strain (Van Keymeulen et al., 2011) with a *Rosa-confetti* reporter (Snippert et al., 2010) (Figure 3C; Table S1). With the same cell kinetics and fate probabilities, we found that the model reliably predicted the experimental observations (Figure 3D), even though animals were exposed to a different tmx-induction regimen. Similarly, as an additional consistency check, we used *Tg(Krt8-CreER); Rosa26R-fGFP* animals (Van Keymeulen et al., 2011) with a low dose of tamoxifen to label scattered luminal cells for up to 8 weeks (Figure 3E). These data were also found to be consistent with the quantitative predictions of our model (Figure 3F; Supplemental Theory).

Our model makes strong predictions about the rates and types of cell division in the tracheal epithelium. We tested these by using nucleotide incorporation assays. We found that BC BrdU incorporation rates provided an estimate of cell cycle times within the range predicted by the model (Supplemental Theory). Moreover, to identify types of cell divisions, we combined 5-ethynyl-2′-deoxyuridine (EdU) incorporation with whole-mount immunostaining to visualize S phase cells (2 hr post-EdU) and their immediate progeny (24 hr post-EdU; Figure 3G; Table S1). The observations confirmed that in wild-type animals, almost 100% of BC divisions result in two BCs, and 95% of SecC divisions result in two SecCs and 5% one SecC and one CC, in excellent agreement with our predictions (Figure 3H; Supplemental Theory). Indeed, if we take the frequency of CC production from SecCs as the lowest 95% confidence interval from our EdU measurements (7% of all SecC divisions) and use the division rate of SecCs from our model, we find that less than 1% of CC production can potentially originate from BLPs (Supplemental Theory). Thus, our EdU experiments support the model prediction that the major route of new CC production at homeostasis is via the SecCs, rather than direct differentiation of the BLPs.

### Single-Cell qRT-PCR Identifies Two Molecularly Distinct Subtypes of BCs

**Figure 4:**
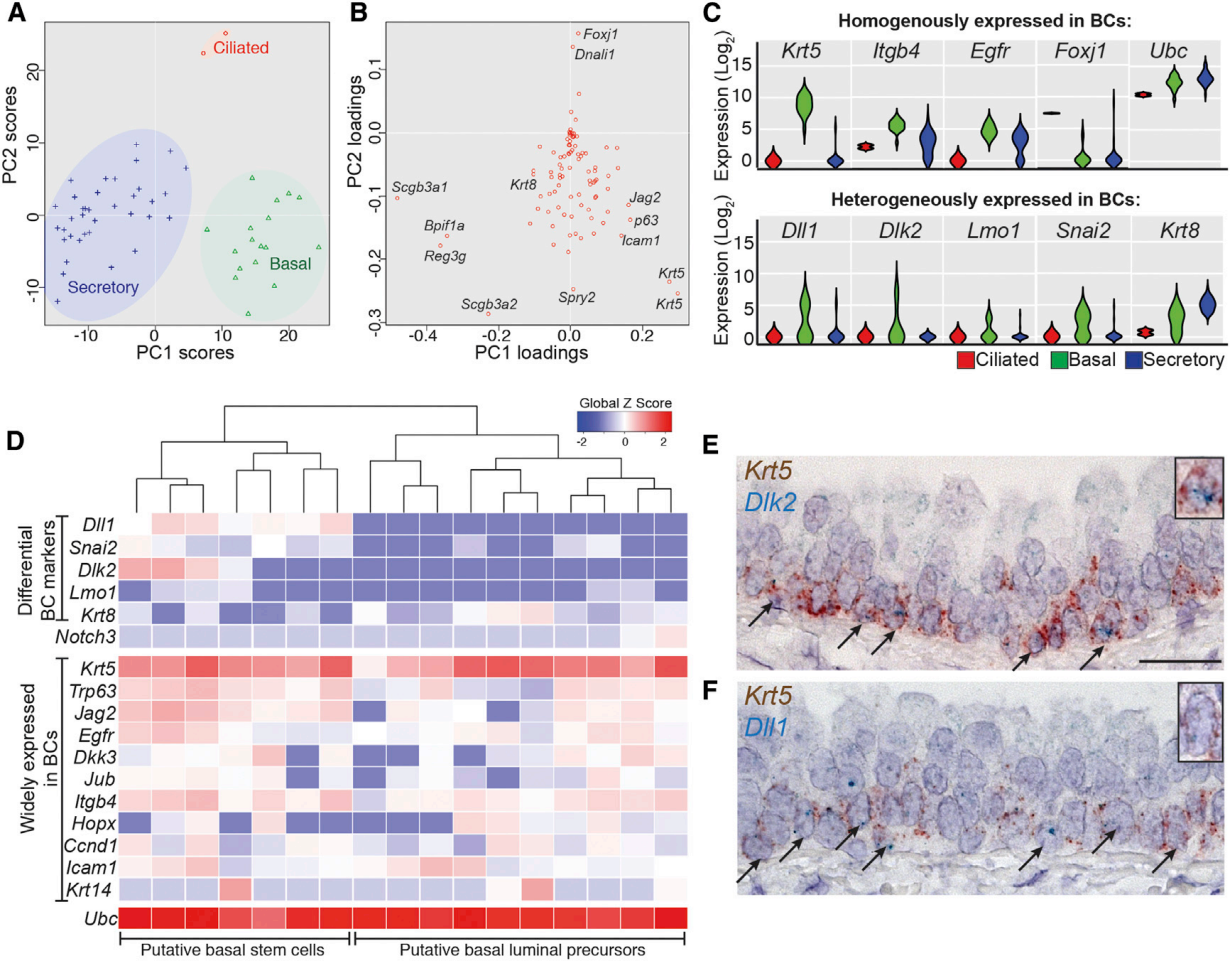
Molecular Analysis Distinguishes Two Subtypes of Basal Cells (A) Principal component analysis (PCA) of 56 single tracheal epithelial cells from the expression levels of all 92 genes determined by qPCR distinguishes between the major cell lineages. (B) Principal component loadings indicate the extent to which each gene contributes to the separation of cells along each component in (A). (C) Violin plots showing log_2_ expression levels of selected genes in BCs (green) or secretory (blue) or ciliated (red) cells. Monophasic (single bulge) plots indicate homogenous gene expression. Biphasic plots (two bulges) indicate two expression levels within the population and show that *Dll1*, *Dlk2*, *Lmo1*, *Snai2*, and *Krt8* are heterogeneously expressed in BCs. Interestingly, *Itgb4* and *Egfr* are heterogeneous within the secretory population but homogenous in the BCs. (D) Heatmap showing unsupervised hierarchical clustering of the BCs only, based on expression levels of 15 of the BC-enriched genes. BCs split into two major groups: putative stem cells and luminal precursors. (E and F) Double mRNA in situ hybridization of wild-type adult trachea sections showing *Krt5* (brown, basal cells) and *Dlk2* (blue, E) or *Dll1* (blue, F). Arrows indicate co-expressing cells. Scale bar, 20 μm. See also Figure S3.

Our experiments support a cellular hierarchy in which there are two distinct BCs. To establish whether these BC populations are molecularly distinct, we performed qRT-PCR on 67 single cells isolated from total epithelium. We analyzed 5 housekeeping, 20 lineage-specific, and 67 genes reportedly enriched in BCs (Hackett et al., 2011; Rock et al., 2009) (Supplemental Experimental Procedures). Cells were grouped by unsupervised hierarchical clustering by using expression levels of all tested genes (Figure S3). This defined three major groups, also seen separated by an independent principal component analysis (Figure 4A). Principal component loadings showed that these represented BCs, SecCs, and CCs (Figure 4B). Similarly, pairwise ANOVA between the groups also showed that each was enriched for expression of definitive markers (Table S2). Analysis of gene expression levels between individual cells within each group showed that *Dlk2*, *Dll1*, *Lmo1*, *Snai2*, and *Krt8* had biphasic patterns within BCs, indicating heterogeneous expression (Figure 4C). This was in agreement with independent unsupervised hierarchical clustering performed on BCs alone (Figure 4D). In this analysis *Dlk2*, *Dll1*, *Lmo1*, and *Snai2* tended to be expressed together in one BC population, while *Krt8* (a luminal cell marker) was more highly expressed in a second population. The only gene previously reported as differentially expressed in BCs, *Krt14*, was detected in a small number of cells in both subpopulations and is therefore unlikely to distinguish them (Figure 4D). mRNA in situ hybridization confirmed the heterogeneous distribution of *Dlk2* and *Dll1* within BCs (Figures 4E and 4F). These data support our model of two distinct BC subpopulations. Moreover, they suggest that one subpopulation is upregulating luminal markers (*Krt8*), and we hypothesized that this is the BLP.

### Low-Level *Krt8* Expression Characterizes a Population of BCs that Are Lost Rapidly from the Tracheal Epithelium

**Figure 5:**
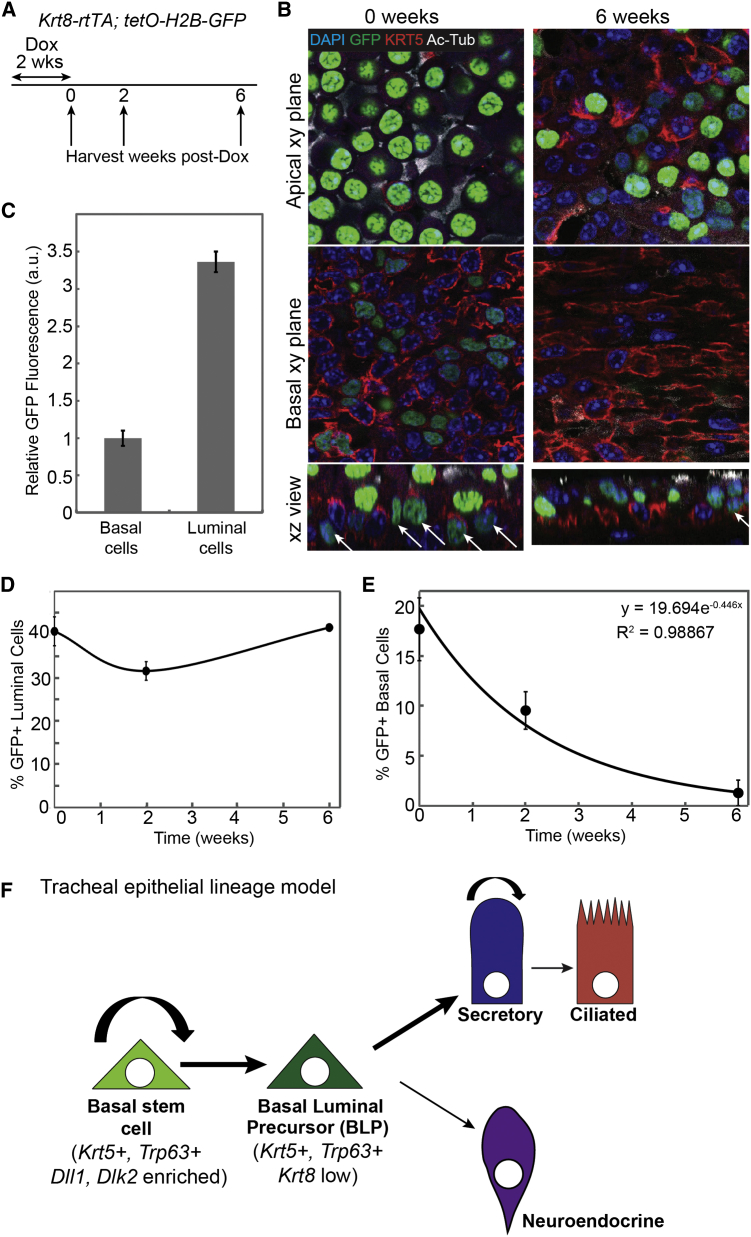
*Krt8* Is Expressed in a Subset of Basal Cells that Are Lost Rapidly from the Basal Layer (A) Schematic of *Tg(Krt8-rtTA); tetO-H2B-GFP* labeling experiment. (B) Representative single confocal xy planes from a confocal stack of a basal area and a more apical area of the whole-mount epithelium with a corresponding xz reconstruction at 0 and 6 weeks post-doxycycline. Blue, Hoechst (nuclei); green, H2B-GFP (short-term lineage label); red, KRT5 (BCs); white, acetylated tubulin (cilia). Scale bar, 10 μm. (C) Plot of the levels of GFP fluorescence in the basal and luminal cells at 0 weeks post-doxycycline. GFP fluorescence intensity was measured in a.u. and the levels were normalized to 1 in the basal cells. Error bars represent 95% confidence intervals. (D) Plot of the percentage of GFP^+^ luminal cells over time. Error bars represent 95% confidence intervals. Note that of the luminal cells, only the SecCs are labeled with GFP, resulting in an ∼40% labeling efficiency of the entire luminal population. (E) Plot of the percentage of GFP^+^ basal cells over time. Error bars represent 95% confidence intervals (n = 3 mice at 0 and 2 weeks; n = 2 mice at 6 weeks). (F) Model of the tracheal epithelial cell lineage. Basal stem cells (BSCs) are enriched in Dl-family ligands and divide, mostly via asymmetric division (94%) to produce on average one new BSC and one BLP. The BLPs upregulate *Krt8* expression and differentiate into luminal cells over the course of ∼11 days. The simplest model is one in which BLPs do not divide. We cannot exclude the possibility of a small number of BLP divisions, although our modeling suggests that any division of BLPs must be occurring at a rate of <50% of the total basal cell divisions, making them unlikely to function as a transit-amplifying progenitor. The terminally differentiated ciliated cells are produced at an extremely low rate at steady state, and our data suggest that division of SecCs is sufficient to account for all new CC production. Hence, BLPs are depicted as differentiating to secretory cell fate. However, it is possible that they also directly produce ciliated cells at a very low frequency. Our data also show that BSCs can directly produce NE cells. Secretory cells divide at a lower rate than BSCs. They have a short half-life and mostly self-renew (>90% self-renewing divisions), but they do generate new ciliated cells at low frequency.

To test the hypothesis that the tracheal epithelium contains a widespread basal *Krt8*^+^ luminal precursor, we used *Krt8-rtTA; tetO-H2B-GFP* animals. Two weeks of exposure to doxycycline labeled ∼18% of BCs and most SecCs (Figures 5A and 5B; Table S1). Significantly, we found that SecC labeling was maintained after a 6-week chase (Figure 5D), whereas BC labeling was almost absent, consistent with our hypothesis (Figure 5E). Moreover, the loss rate of the GFP^+^ BCs fitted extremely well to an exponential decay curve (R^2^ = 0.998867) giving a measured loss rate of the BLPs of 16 days, in good agreement with our predicted rate of 11 ± (7, 4) days. Immediately after induction, labeled BCs had 3-fold lower levels of GFP than luminal cells (Figure 5C; Table S1), consistent with a lower expression level of *Krt8* and further supporting our hypothesis that they are a distinct, differentiating BC subpopulation. These data are consistent with our model of two BC subpopulations, one of which is fated as a luminal precursor (Figure 5F).

## Discussion

Uncovering the proliferative hierarchy, quantitative fate behavior, and molecular profile of a slow-cycling tissue inevitably requires detailed and long-term studies. By combining the results of long-term lineage tracing using multiple drivers with single-cell gene expression profiling, we have shown that tracheal KRT5^+^ BCs include two subpopulations: stem cells (BSCs) and luminal precursors (BLPs). BSCs maintain epithelial homeostasis by dividing in a balanced manner to self-renew and produce BLPs. BLPs are widespread, long-lived (∼2 weeks) luminal precursors, which upregulate the luminal cytokeratin *Krt8* before overt signs of differentiation (Figure 5F). Our data suggest that these cells do not function as a traditionally defined transit-amplifying population. Rather, the existence of a widely distributed, luminal-fated, long-lived precursor, which is morphologically indistinguishable from the stem cell, is a surprising finding and has significant implications for fundamental and reparative biology, and disease initiation, in the airways. In particular, our model suggests that there must be at least two separate signaling events controlling luminal cell differentiation: specification as a BLP (which may or may not be concurrent with secretory/ciliated specification), and, separately, maturation of that cell into a differentiated luminal cell. We have therefore defined another event (maturation) at which normal homeostasis can be perturbed in disease.

A KRT5^+^, KRT8^+^ parabasal cell population was recently detected in wild-type adults (∼25% of the total BC population). The authors suggested that these cells were precursors of the luminal cells and showed that their abundance is regulated by NOTCH3 signaling (Mori et al., 2015). Interestingly, we observed higher *Notch3* mRNA expression in a small number of BLPs that also expressed *Krt8* (Figure 4D). It is likely that the parabasal cells and BLPs (which we propose comprise ∼50% of the total BC population) are overlapping cell populations, but their exact relationship is yet to be defined.

Our conclusion that BSCs maintain the airways by a process of stochastic homeostasis is in principle similar to the model defined for the BC population in the mouse inter-follicular epidermis (Clayton et al., 2007). This means that each time a BSC divides, it has a certain probability of making a symmetric or asymmetric division (Figure 2H). The implication of this finding is that an individual BSC can by chance make a large number of symmetric self-renewing divisions (compensated for, at a population level, by symmetric differentiation divisions of other BSCs) leading to neutral drift in the population of clones. This is consistent with the model proposed for human airway BSCs in vivo based on clonal analysis of naturally occurring mitochondrial mutations (Teixeira et al., 2013). Clonal experiments in mice provide us with richer datasets than those available in human samples due to the defined time courses and greater numbers of replicates. This has allowed us to greatly extend the observations of Teixeira et al. and identify the BLPs, the rapid rate of SecC turnover, and the production of NE cells by BSCs. In the future, it will be important to test if these observations are also recapitulated in human airways.

The KRT5^+^ BSC that we characterize achieves perfect self-renewal for at least 17 months. We cannot exclude the existence of an additional, rarely dividing, minority BSC in the trachea (Borthwick et al., 2001). However, our data show that such a cell is not required for homeostatic turnover. A subset (<20%) of BCs express KRT14. These cells typically occur in clusters and have been proposed to be unipotent stem cells at steady state (Ghosh et al., 2011). We detected rare *Krt14*^*+*^ cells in both the BSC and BLP populations, suggesting that KRT14 is not a reliable marker of stem cell identity. Previous *Tg*(*KRT14-CreER)* lineage labeling studies in which unipotent BC clones were observed used a time course of ∼40 days (Ghosh et al., 2011; Hong et al., 2004) and may not have allowed sufficient time for BLPs to differentiate into luminal cells. We speculate that the clustered distribution of KRT14^+^ BCs and the rapid upregulation of KRT14 levels post-injury indicate a structural role for this intermediate filament protein.

The trachea does have an additional dividing cell population at steady state: the SecC. We confirm that SecC to BC reversion does not happen at homeostasis (Rawlins et al., 2009). Moreover, we show that SecCs have a short half-life compared to CCs, are not traditionally defined transit-amplifying cells but do preferentially self-renew, such that at any given time, ∼44% of SecCs within the epithelium are derived from a pre-existing SecC. This is analogous to prostate and breast epithelia (Chua et al., 2014; Ousset et al., 2012; Rios et al., 2014; Van Keymeulen et al., 2011) where luminal progenitors are recognized as a cell of origin for some cancers (Bai et al., 2013; Molyneux et al., 2010; Wang et al., 2013, 2014). Thus, we may need to consider multiple potential initiating cells for airway carcinogenesis.

Why does the tracheal epithelium contain a widespread, long-lived luminal precursor population that is located in the basal position? We speculate that post-injury, BLPs may either rapidly differentiate or revert to stem cell fate and thus function as a basally positioned reserve progenitor that is not exposed to inhaled agents. Significantly, a recent publication has identified a population of steady-state BCs (∼12% of the total) that express low levels of transcription factors usually found in more differentiated luminal cells. Rarely, these BCs were also KRT8^+^ (Pardo-Saganta et al., 2015). Post-injury, the abundance of luminal transcription factor-positive BCs increased to ∼50% of the total BCs, and they were subsequently observed to proliferate. Our model has BLPs present as ∼50% of the total steady-state BC population. It is highly likely that the ∼12% of transcription-factor-positive homeostatic BCs identified by Pardo-Saganta et al. (2015) correspond to the most differentiated of the homeostatic BLPs that we identified. If this hypothesis is correct, the post-injury role of BLPs is indeed to both rapidly differentiate and proliferate. However, exact testing of this hypothesis will require more specific tools for lineage-labeling the BLP population. Given that the BLPs are a subpopulation of BCs that, at homeostasis, are undergoing a slow differentiation process to luminal fate, a unique transcription factor or other specific molecular marker may be hard to find.

Are BLPs a unique aspect of airway stem cell biology, or could other epithelia also contain fate-committed cells that are morphologically indistinguishable from the stem cells? A recent report using prospective isolation techniques suggests that there is a hierarchy of BC organization in the mouse esophagus, with stem cells being molecularly distinct from more committed suprabasal precursors (DeWard et al., 2014). Similarly, we note that the enteroblast (EB) cell in the *Drosophila* midgut can only be distinguished from the stem cell by expression of *Dl* (Micchelli and Perrimon, 2006; Ohlstein and Spradling, 2006, 2007) and occupies a similar position in the cellular hierarchy to tracheal BLPs. We therefore speculate that uncoupling division of the stem/progenitor from morphological differentiation of the progeny may be a previously undetected general phenomenon in epithelia, with steady-state rates of cell maturation being controlled by cell-type-specific mechanisms. Our strategy of long-term in vivo lineage tracing, coupled with single-cell molecular analysis, should prove widely applicable for the fine dissection of homeostatic lineage potential in epithelia such as the esophagus, prostate, mammary gland, and skin, where no obvious morphological stem cell niches exist.

## Experimental Procedures

See Supplemental Experimental Procedures for a full description of all materials and methods used.

### Animals

All experiments were performed under license PPL80/2326. *Tg(KRT5-CreER)* transgenic (Rock et al., 2009), *Krt5-CreER*^*T2*^ knockin (Van Keymeulen et al., 2011), *Scgb1a1-CreER* (Rawlins et al., 2009), *Tg(Krt8-CreER)* (Van Keymeulen et al., 2011), *Rosa26R-fGFP* (Rawlins et al., 2009), *Rosa-confetti* (Snippert et al., 2010), and *tetO-H2B-GFP* (Tumbar et al., 2004) mice have been described. *Krt8-rtTA* transgenic mice were generated by using a fragment of the murine *Krt8* gene. Males and females >8 weeks old were used in all experiments. Wild-type mice were C57Bl/6J.

### Lineage Tracing

Low-frequency activation of the reporter was achieved by a single intraperitoneal injection of tamoxifen (Sigma-Aldrich, T5648) at a dose of 25 μg/g body weight in *Tg*(*KRT5-CreER); Rosa26R-fGFP* or 13 μg/g body weight in *Tg*(*Krt8-CreER); Rosa26R-fGFP* mice or with two doses of 5 mg tmx per mouse spaced 48 hr apart in *Krt5*^*CreER/+*^*; Rosa-confetti* mice. Doxycycline was administered to *Krt8-rtTA; tetO-H2B-GFP* mice in food at a dose of 10 g/kg (SAFE-DIETS) for 2 weeks. BrdU was given intraperitoneally at 30 μg/g body weight and EdU at 50 μg per mouse.

### Whole-Mount Immunostaining

For *Rosa26R-fGFP* and wild-type mice, tracheas were fixed overnight in 4% paraformaldehyde at 4°C. Primary antibodies were anti-GFP (chicken, 1:1,000; Abcam, AB13970), anti-KRT5 (rabbit, 1:500; Covance, PRB-160P), anti-acetylated tubulin (mouse, 1:1,000; Sigma, T7451), and anti-PGP9.5 (guinea pig, 1:500; Neuromics, GP14104). Secondary antibodies were Alexa Fluor conjugates (Life Technologies, 1:2,000). Samples were processed to 97% TDE (2′2′-thiodiethanol) for mounting. For mice carrying *tetO-H2B-GFP* or *Rosa26R-confetti*, the whole-mount protocol was adapted to enable direct visualization of native fluorescence. Anti-GFP staining was omitted, and samples were mounted in Glycergel (Dako) + 2.5% DABCO.

### Section Immunostaining

8- to 10-μm cryosections were stained with: anti-acetylated tubulin (mouse, 1:1,000; Sigma, T7451), anti-BrdU (mouse, 1:500; Sigma, B8434), anti-Ecad (rat, 1:3000; Life Technologies, 13-1900), anti-Krt5 (rabbit, 1:500; Covance, PRB-160P), anti-T1α (1:1,000; DSHB, 8.1.1), and anti-Scgb1a1 (rabbit, 1:500; Santa Cruz, sc25555). Antigen retrieval was used for BrdU (2 N HCl 30 min 37°C, 0.5% trypsin 5 min, room temperature).

### mRNA In Situ Hybridization

Trachea were formalin-fixed for 24 hr at room temperature and paraffin embedded. 5-μm sections were processed for RNA in situ with the RNA Scope 2-plex Detection Kit (Chromogenic) according to the manufacturer’s standard protocol (Advanced Cell Diagnostics). RNAscope probes were *Krt5* (NM 027011.2, region 666–2,086), *Dlk2* (NM 023932.3, region 267–1,279), and *Dll1* (NM 007865.3, region 888–1,883).

### Microscopy and Image Scoring

z stacks of the full epithelial thickness were acquired at an optical resolution of 1,024 × 1,024 with an optical z slice every 1 μm. Clones were scored manually by looking through the entire z depth of the tracheal epithelium in FV viewer or LAS AF software to score the identity of all labeled cells. For *tetO-H2B-GFP* samples, z stacks were acquired at an optical resolution of 1,024 × 1,024, with a z slice every 0.38 μm. Fluorescence intensity was assessed in Fiji, using the Gurdon Institute Imaging Facility’s plugin, ObjectScan.

Cryosections for analysis of cellular composition/density were imaged on an Olympus FV1000, using a 100× oil objective (numerical aperture [NA] 1.4). The length of the basement membrane in each image was measured in Fiji. Density was calculated as the number of cells present per μm of basement membrane. Cryosections for BrdU analysis were imaged on a Zeiss AxioImager compound microscope, using a 20× air objective (NA 0.8) and counted in Fiji.

### Single-Cell qRT-PCR

The distal tracheal epithelium was peeled away from the underlying mesenchyme following a brief dispase digest and dissociated to single cells as described previously (Rock et al., 2009). Unsorted epithelial cells were loaded into a Fluidigm C1 machine on a 10- to 17-μm chip at a concentration of ∼400 cells/μl for cell capture, lysis, cDNA synthesis, and target pre-amplification. 67 single cells were used for subsequent qRT-PCR on a 96.96 Fluidigm Dynamic array using a Biomark qPCR machine using TaqMan gene expression assays (Life Technologies). Data analysis was performed in the Fluidigm Singular Analysis Toolset 3.0 in R.

### Modeling

See Supplemental Experimental Procedures and Supplemental Theory for all details of model construction.

## Author Contributions

J.K.W. designed and performed experiments, analyzed data, and wrote the manuscript. S.R. designed and executed the biophysical modeling and wrote the manuscript. A.C.W. and B.G. assisted with qRT-PCR experimental design and analysis and edited the manuscript. A.W., M.O., A.V.K., and C.B. provided mice and trachea samples and assisted with experimental design. B.D.S. led the biophysical modeling and edited the paper. E.L.R. conceived and led the project, performed experiments, analyzed data, and wrote and edited the manuscript.

## Supplemental Information

Document S1. Supplemental Experimental Procedures, Supplemental Theory, Figures S1–S3, and Table S2

Table S1. Raw Data for All of the Clonal Analysis, the EdU Data Set, and the K8-H2B-GFP, Related to Figures 1, 2, 3, and 5

Document S2. Article plus Supplemental Information
