# Supplementary material for: Clonal Dynamics Reveal Two Distinct Populations of Basal Cells in Slow-Turnover Airway Epithelium
Source: Cell Rep. 2015 Jun 25;12(1):90–101. doi: 10.1016/j.celrep.2015.06.011 (PMC4518462; doi:10.1016/j.celrep.2015.06.011)
Supplement: Document S1. Supplemental Experimental Procedures, Supplemental Theory, Figures S1–S3, and Table S2 [file mmc1.pdf]

Cell Reports
Supplemental Information

Clonal Dynamics Reveal Two Distinct Populations of Basal Cells in Slow-Turnover Airway Epithelium

Julie K. Watson, Steffen Rulands, Adam C. Wilkinson, Aline Wuidart, Marielle Ousset, Alexandra Van Keymeulen, Berthold Göttgens, Cédric Blanpain, Benjamin D. Simons, and Emma L. Rawlins

Figure S1, related to Figure 1. Analysis of cell types, proliferation rates and density indicates that the tracheal epithelium is at homeostasis for most of the period under study. (A) Timecourse of experiment. Animals used in homeostasis experiments were all *Tg(KRT5-CreER); Rosa26R-fGFP* and > 8 weeks old at time of tmx exposure. (B) Representative image of epithelium from BrdU incorporation experiment. Red: KRT5 (basal cells), green: BrdU (cell in S-phase). (C, D) Histograms depicting the proportion of (C) basal and (D) secretory cells that have incorporated BrdU following a 2-hour pulse, at 0.5, 12 and 52 weeks in the dorsal tracheal epithelium adjacent to the dorsal longitudinal muscle (blue), or the cartilage (red). (E-J) Representative images of epithelia from cellular composition and density experiments. (E) 0.5 weeks, (F) 3 weeks, (G) 52 weeks adjacent to the longitudinal smooth muscle. (H) 0.5 weeks, (I) 3 weeks, (J) 52 weeks adjacent to the cartilage/inter-cartilage regions of mesenchyme. White: T1α (basal cells); purple: Scgb1a1 (secretory cells); blue: acetylated-tubulin (ciliated cells); green: E-cadherin (lateral cell membranes). (K, L) Histogram depicting (K) the cellular composition and (L) cellular density of the epithelium at 0.5, 12 and 52 weeks, in muscle and cartilage regions. (M) Plot of the mean length of the trachea from the carina to the 6th cartilage ring over time. (N) Plot of the mean width of the trachea at the 3rd cartilage ring above the carina over time. All error bars = 95% confidence intervals. Scale bars = 10μm. See Table S1 for all raw data.

Figure S2, related to Figure 1. Attempts to stratify the *Tg(KRT5-CreER); Rosa26R-fGFP* basal cell clonal data indicate that the dorsal-distal mouse trachea is a homogenous region with one epithelial cell hierarchy. (A) Plot of the mean number of total cells per clone, split into male (blue) and female (red) mice. (B) Plot of the mean number of total cells per clone, with the data split into 3 broad proximal-distal regions based on distance from the carina. Most distal (blue), 2nd most distal (red), 3rd most distal (green). (C) Plot of the mean number of total cells per clone, split into clones over the dorsal longitudinal muscle (blue), or over the cartilage region (red). (D, E) Plot of the mean number of cells of each type per clone (green = basal; blue = secretory; red = ciliated), split into

clones located above dorsal longitudinal muscle (D) and clones located above the cartilage region (E). The graphs in (C-E) suggest that there are small systematic differences in the behaviour of the clones located over the dorsal muscle versus the cartilage, but this was not statistically significant using a Mann-Witney U-test (Supplemental Theory). Error bars in all plots = 95% confidence intervals, with n set to the number of mice.

Figure S3, related to Figure 4. Unsupervised hierarchical clustering of all single tracheal epithelial cells which passed quality controls, based on 96 independent qPCR reactions covering 92 genes. The three clusters of cells are labelled ciliated, basal and secretory. These identities were determined by mapping back onto an independent principal component analysis (see Figure 4A, B), and also by pair-wise ANOVA between each group which showed that each group was enriched for definitive markers of the basal, secretory or ciliated epithelial cells (see Table S3).

Table S1, related to Figures 1, 2, 3 and 5. Raw data for all of the clonal analysis, the EdU data set and the *K8-H2B-GFP*.

Table S2, related to Figures 4 and S3. Genes differentially expressed between the three major tracheal epithelial cell groups based on pairwise ANOVA analysis of single cell RT-qPCR data.

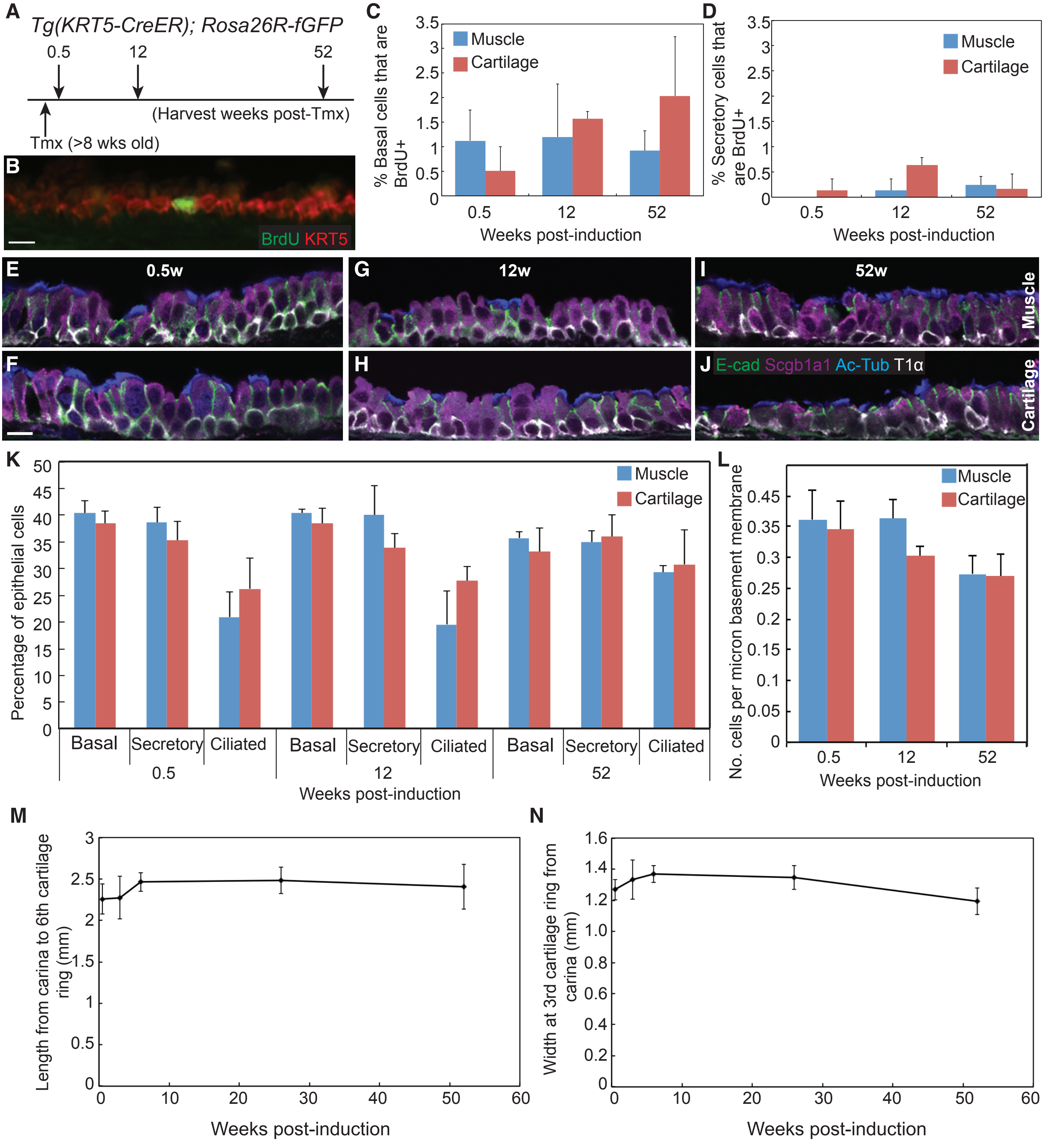

Watson et al. Sup Fig 1

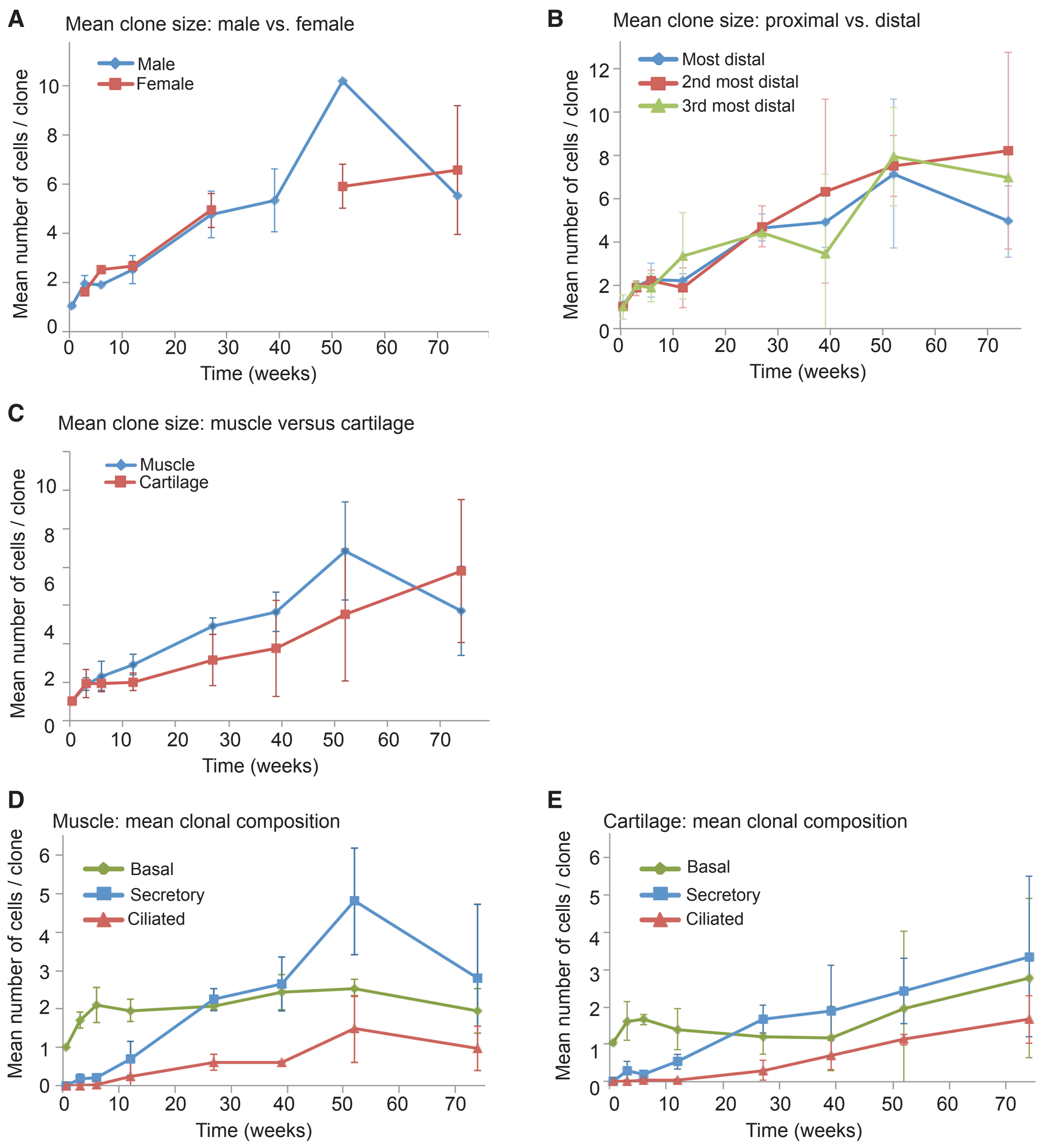

Supplementary Fig. 2 Watson et al.

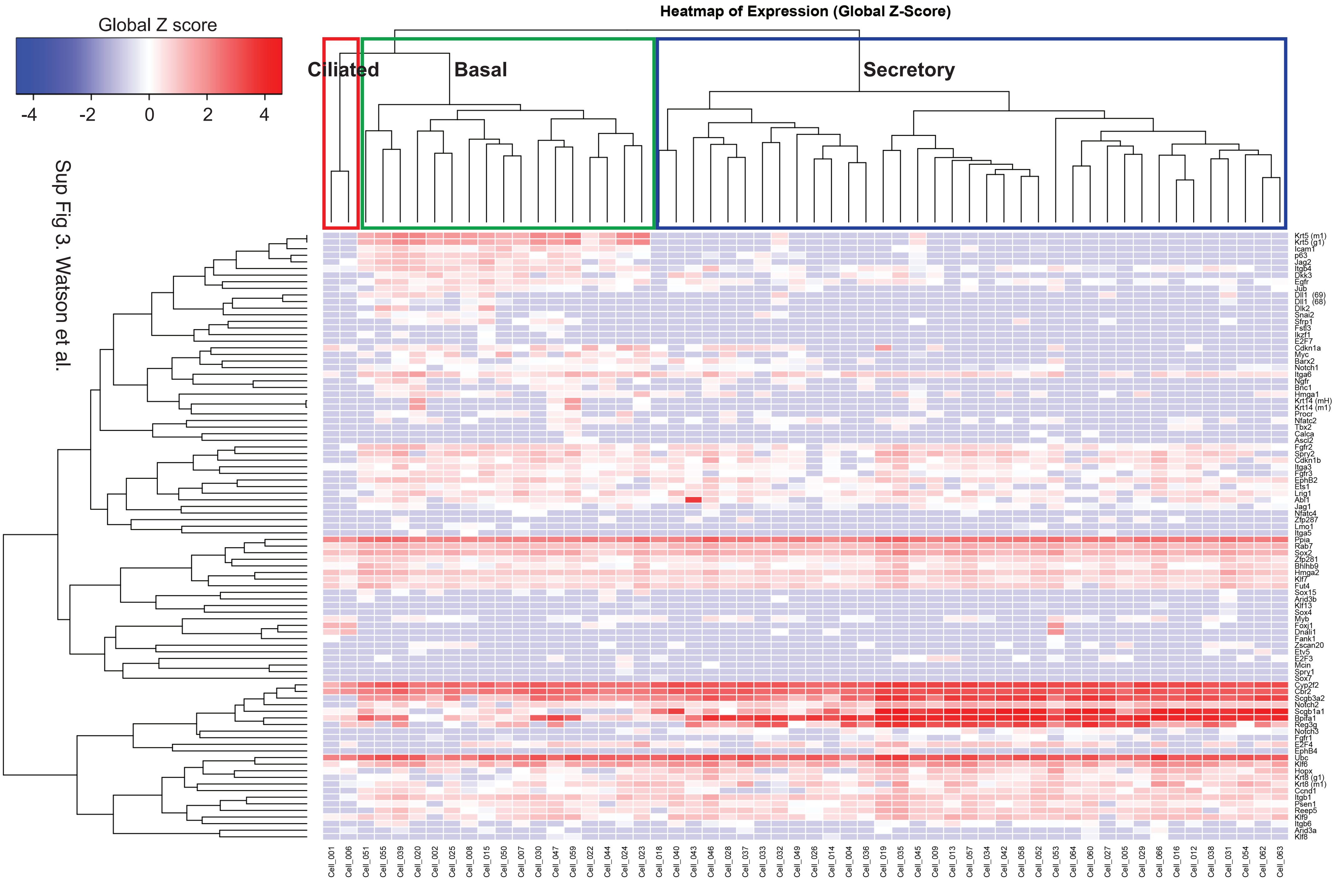

Sup Fig 3. Watson et al.

Table S2. Genes differentially expressed between the three major tracheal epithelial cell groups based on pairwise ANOVA analysis of single cell RT-qPCR data. ANOVA was used to compare the mean Log_2 expression level of each gene between the basal, secretory and ciliated cell groups defined in Figure 4A. Genes with an ANOVA p value <0.05 and a >1.2x Log_2 fold-change in mean expression level between two of the cell groups are listed.

**Basal cell population-enriched transcripts** (p value from comparison of BC vs. secretory populations)	**Secretory cell population-enriched transcripts** (p value from comparison of BC vs. secretory populations)	**Ciliated cell population-enriched transcripts** (p value from comparison of BC vs. ciliated populations)
*Krt5** (0)	*Bpifla* (2.08×10^{-11})	*Foxj1** (1.83×10^{-7})
*Trp63** (0)	*Scgb1a1* (1.78×10^{-10})	*Dnai1** (2.64×10^{-7})
*Jag2** (0)	*Cyp2f2* (3.93×10^{-9})	*Fank1** (2.30×10^{-4})
*Icam1** (0)	*Krt8* (1.19×10^{-7})	
*Myc** (1.10×10^{-7})	*Cbr2* (1.54×10^{-7})	
Itgb4 (2.84×10^{-7})	*Reg3g* (2.18×10^{-7})	
Notch1 (1.92×10^{-6})	*Scgb3a2* (1.19×10^{-6})	
*Ngfr** (2.22×10^{-6})	*Notch2* (1.29×10^{-5})	
*Jub** (4.81×10^{-6})	*Notch3* (6.42×10^{-5})	
*Snai2** (2.87×10^{-5})	*Hopx* (2.12×10^{-3})	
Fgfr2 (5.2×10^{-5})	*Fgfr1* (3.02×10^{-2})	
*Sfrp1** (6.58×10^{-5})		
Egfr (1.09×10^{-4})		
Fgfr3 (1.49×10^{-4})		
*Procr** (1.75×10^{-4})		
*Sox15** (2.81×10^{-4})		
Dkk3 (3.27×10^{-4})		
Cdkn1a (7.84×10^{-4})		
*Dlk2** (5.91×10^{-3})		
Barx2 (6.08×10^{-3})		
Hmga1 (7.07×10^{-3})		
*Bnc1** (8.05×10^{-3})		
*Dll1** (9.16×10^{-3})		
*Krt14** (2.56×10^{-2})		
Itga6 (3.76×10^{-2})		
*Lmo1** (2.32×10^{-2})		

EXPERIMENTAL PROCEDURES

Animals. All experiments were approved by University of Cambridge local ethical review committees and conducted according to Home Office project license PPL80/2326. Mouse strains *Tg(KRT5-CreER)* transgenic (Rock et al., 2009), *Krt5-CreER^{T2}* knock-in (Van Keymeulen et al., 2011)*, Scgb1a1-CreER* (Rawlins et al., 2009), *Tg(Krt8-CreER)* (Van Keymeulen et al., 2011), *Rosa26R-fGFP* (Rawlins et al., 2009), *Rosa-confetti (Snippert et al., 2010)*, *tetO-H2B-GFP* (Tumbar et al., 2004) have been described previously. Strains used for long-term lineage-tracing: *Tg*(*KRT5-CreER)*, *Scgb1a1-CreER*, *Rosa26R-fGFP* were all maintained on a C57Bl/6J inbred background; other strains were at least N2 C57Bl/6J. All animals used for experiments were >8 weeks old. For transgenic experiments a mixture of males and females were used and no gender-specific differences observed. Wildtype C57Bl/6J inbred mice were used for wholemount EdU analysis, mRNA in situ hybridisation and single cell RT-qPCR.

Generation of *Krt8-rtTA* mice. The rtTA fragment from pTetON Advanced plasmid preceded by the β-globin intron and followed by a SV40 polyA signal was subcloned into pBluescript II SK+. The 3.5-kb sequence upstream of the ATG codon of the murine *Krt8* gene, obtained from the BAC clone RO-254K21 (BACPAC Resources Center, Children's Hospital Oakland Research Institute) using the forward primer 5'-GGTGGATCACTTGCCCCCTCCGTTTG-3' and the reverse primer 5'-GGGACAGCGCCCAGCGAAGGCCC-3', was cloned upstream of the β-globin intron. The resulting *Krt8-rtTA* fragment of 5.1 kb was released from the backbone by NotI and KpnI digestion and was microinjected into fertilized oocytes to generate transgenic mice (in the transgenic facility of the Université catholique de Louvain, Brussels, Belgium). Seven transgenic founders were first identified by PCR, out of 42 mice born. Expression profiles of

the *Krt8-rtTA* founders were screened with reporter *tetO-H2B-GFP* mice. Five founders expressed GFP in cells expressing the endogenous *Krt8*, and one founder was used throughout this study.

Lineage-tracing. Low frequency activation of the reporter allele was achieved by a single intraperitoneal injection of tamoxifen (Sigma, T5648) dissolved in vegetable oil at a dose of 25 µg per gram body weight in *Tg*(*KRT5-CreER); Rosa26R-fGFP* mice, or 13 µg per gram body weight in *Tg*(*Krt8-CreER); Rosa26R-fGFP* mice, or with 2 intraperitoneal doses of 5mg tmx per mouse spaced 48h apart in *Krt5*$^{CreER/+}$*; Rosa-confetti*. Doxycycline was administered to *Krt8-rtTA; tetO-H2B-GFP* mice in food at a dose of 10g/kg (SAFE-DIETS) for 2 weeks followed by chase periods of up to 6 weeks.

BrdU/EdU Labelling. BrdU was administered intraperitoneally at a dose of 30 µg/g body weight and tracheas harvested 2 hours post-injection. EdU was administered by intraperitoneal injection at a dose of 50 µg per mouse and tracheas were harvested at 2 hours or 24 hours post-injection. EdU was detected using the Click-it EdU kit (Life Technologies) in wholemount preparations.

Wholemount immunostaining

For mice carrying the *Rosa26R-fGFP* locus and wildtype mice, dissected tracheas were fixed overnight in 4% paraformaldehyde at 4°C followed by PBS washes. Blood vessels and connective tissue were removed manually and trachea were cut into dorsal and ventral halves longitudinally. Tracheal halves were permeabilized in 1% Triton X-100 in PBS for 1 hour at room temperature, followed by PBS washes, blocking overnight, and primary antibody incubation for 2 nights at 4°C in block (block: 1% BSA, 5% Normal Donkey Serum, 0.2%

Triton X-100 in PBS). Washes were performed in PBTx (PBS, 0.2% Triton X-100) and secondary antibodies incubated for 2 nights at 4°C in 5% Normal Donkey Serum, 0.2% Triton X-100 in PBS. DAPI (4',6-diamidino-2-phenylidole) or Hoecsht were diluted 1:5,000 in 0.2% Triton X-100 in PBS and incubated for 1 hour at room temperature followed by 0.2% Triton X-100 in PBS washes. Samples were then washed in PBS followed by incubation in increasing concentrations (10%, 25%, 50% for 2h each and 97% overnight) of TDE (2'2'-thiodiethanol) in 0.25x PBS at 4°C and mounting in 97% TDE under a raised coverslip. The following primary antibodies were used: anti-GFP (chicken, 1:1000, Abcam, AB13970), anti-KRT5 (rabbit, 1:500, Covance, PRB-160P), anti-acetylated tubulin (mouse, 1:1000, Sigma, T7451), anti-Pgp9.5 (guinea pig, Neuromics, GP14104). All secondary antibodies were Alexa Fluor conjugated from Life Technologies and used at 1:2000 dilution.

For mice carrying *tetO-H2B-GFP* or *Rosa26R-confetti* loci, the wholemount protocol was adapted to enable direct visualisation of native fluorescence. Anti-GFP staining was omitted, and samples were mounted in Glycergel (Dako) + 2.5% DABCO , rather than TDE. For *Rosa26R-confetti* samples, anti-acetylated-tubulin was detected using an anti-mouse Alexafluor-405 secondary antibody (1:200, Jackson Immunoresearch), anti-rabbit KRT5 with an Alexafluor-647 secondary antibody (Life Technologies, 1:2000) and DAPI/Hoechst staining was omitted.

Section Immunostaining

For cryoembedding tracheas were fixed in 4% paraformaldehyde at 4°C for 4 hours. Samples were washed in PBS, sucrose protected, embedded in OCT (Optimum Cutting Temperature Compound, Tissue Tek) and sectioned at 8-10 μm. The following primary antibodies were used: anti-acetylated tubulin (mouse, 1:1000, Sigma, T7451), anti-BrdU (mouse, 1:500,

Sigma, B8434), anti-Ecad (rat, 1:3000, Life Technologies, 13-1900), anti-Krt5 (rabbit, 1:500, Covance, PRB-160P), anti-T1α (1:1000, DSHB, 8.1.1), anti-Scgb1a1 (rabbit, 1:500, Santa Cruz, sc25555). Antigen retrieval was used for BrdU staining by warming samples to 37°C in 2N HCl for 30 minutes, and then incubating in 0.5% trypsin for 5 minutes at room temperature. All secondary antibodies were Alexa Fluor conjugated from Life Technologies and used at 1:2000 dilution. Slides were mounted in Fluoromount (Sigma).

mRNA in situ hybridisation

Trachea were formalin-fixed 24h at room temperature and processed for paraffin embedding via an ethanol gradient. Tissue sections were cut at 5 μm and processed for RNA in situ detection using the RNA Scope 2-plex Detection Kit (Chromogenic) according to the manufacturer's standard protocol (Advanced Cell Diagnostics). RNAscope probes used were: *Krt5* (NM 027011.2, region 666-2086) which was detected using the Fast Red detection reagent; and *Dlk2* (NM 023932.3, region 267-1279) and *Dll1* (NM 007865.3, region 888-1883) which were detected using the Green detection reagent.

Microscopy and Image scoring

For wholemount samples from mice carrying the *Rosa26R-fGFP* or *Rosa26R-confetti* locus, the study area was first imaged using a 20x lens to identify the locations of clones. Images of clones were then acquired using a 60x oil objective (NA 1.35 or 1.4) on either an Olympus FV1000, or a Leica Sp8. Z-stacks of the full epithelial thickness were acquired at an optical resolution of 1,024 x 1,024 with an optical z slice every 1 μm. Clones were scored manually by looking through the entire z depth of the tracheal epithelium in FV viewer or LAS AF software to score the identity of all labelled cells. Cells were scored based on the following criteria: basal cells – KRT5^{+}; ciliated cells – acetylated tubulin^{+} cilia on their apical surface;

secretory cells – luminal cells with no cilia, in addition auto-fluorescent secretory granules were present in these cells. (This definition of secretory cells means that any labelled brush or neuroendocrine cells, which are present in the trachea at low numbers and can only be distinguished by specific staining, would have been scored as secretory). Cells were scored as within 1 clone if they were within <1 cell diameter of each other.

For wholemount samples from mice carrying the *tetO-H2B-GFP* locus, randomly selected locations within the study area were imaged using a 100x oil objective (NA 1.4) on a Leica SP8. Z-stacks were acquired at an optical resolution of 1,024 x 1,024, with a z-slice every 0.38 μm. Flourescence intensity was assessed in Fiji, using the Gurdon Institute Imaging Facility's plugin, ObjectScan. Nuclei were defined based on Hoechst staining, and the total GFP signal within the nuclear volume measured. Object maps were checked manually to exclude any nuclei that were mapped incorrectly. Cell types were scored as for the clonal analysis experiments, but with GFP positivity indicating KRT8 expression.

For wholemount samples from wildtype mice used for EdU analysis, the study area was imaged on an Olympus FV1000, using a 40x oil objective (NA 1.3). Z-stacks were acquired at an optical resolution of 1,024 x 1,024, with a z-slice every 1 μm. Cell types were scored as for the clonal analysis experiments, but with EdU incorporation marking cells in S-phase, or their immediate progeny.

Cryosections used for analysis of cellular composition and density were imaged on an Olympus FV1000, using a 100x oil objective (NA 1.4). Z-stacks were acquired at an optical resolution of 1,024 x 1,024, with a z-slice every 0.45 μm. The numbers of cells of each type were assessed using FV1000 software. Cells were scored based on the following criteria:

basal cells – T1α^+; ciliated cells – acetylated tubulin$^+$ cilia on their apical surface; secretory cells – Scgb1a1$^+$ cells. The length of the basement membrane in each image was measured in Fiji. Density was calculated as the number of cells present per μm of basement membrane.

Cryosections used for BrdU analysis were captured on a Zeiss AxioImager compound microscope, using a 20x air objective (NA 0.8) and counted in Fiji. Cells were scored based on the following criteria: basal cells in S-phase – KRT5$^+$BrdU$^+$; basal cells not in S-phase – KRT5$^+$BrdU$^-$; secretory cells in S-phase – KRT5$^-$BrdU$^+$. The total number of secretory cells was not counted directly but was inferred as being equal to the number of basal cells, based on the cellular composition experiments, which were performed on tracheal sections from the same mice.

Single cell RT-qPCR. The distal trachea was isolated (from the carina up to ~5th cartilage ring), the epithelium was peeled away from the underlying mesenchyme following a brief dispase digest and dissociated to single cells as described (Rock et al., 2009). Unsorted epithelial cells were loaded into a Fluidigm C1 machine on a medium (10-17 μm) chip at a concentration of ~400 cells/μl for cell capture, lysis, cDNA synthesis and target pre-amplification. 67 single cells were used for subsequent RT-qPCR on a 96.96 Fluidigm Dynamic array using a Biomark qPCR machine. Taqman gene expression assays (Life Technologies) were used for expression analysis (Supplemental Experimental Procedures). Data analysis was performed in the Fluidigm Singular Analysis Toolset 3.0 in R. Briefly, cells with aberrant transcript levels were excluded using the Outlier analysis function based on the median levels of 23 highly expressed genes, and also by manual curation based on expression of <4 of the 5 housekeeping genes included in the analysis. 56 single cells passed

these quality controls and Principle Component Analysis, unsupervised hierarchical clustering, violin plots and ANOVAs were all performed using Singular.

Modelling. See theoretical supplement for all details of model construction. Note that for the analysis of the EdU data (Figure 3H) only single EdU^+ cells at 2 hours and pairs of EdU^+ cells at 24 hours were included in the comparison to the model. The full data set included some groups of >2 cells and some single cells at the 24 hour time point, likely resulting from clone merging or EdU toxicity respectively. The complete data set is available in Table S1.

Rawlins, E.L., Okubo, T., Xue, Y., Brass, D.M., Auten, R.L., Hasegawa, H., Wang, F., and Hogan, B.L. (2009). The role of Scgb1a1+ Clara cells in the long-term maintenance and repair of lung airway, but not alveolar, epithelium. Cell stem cell *4*, 525-534.

Rock, J.R., Onaitis, M.W., Rawlins, E.L., Lu, Y., Clark, C.P., Xue, Y., Randell, S.H., and Hogan, B.L. (2009). Basal cells as stem cells of the mouse trachea and human airway epithelium. Proc Natl Acad Sci U S A *106*, 12771-12775.

Snippert, H.J., van der Flier, L.G., Sato, T., van Es, J.H., van den Born, M., Kroon-Veenboer, C., Barker, N., Klein, A.M., van Rheenen, J., Simons, B.D.*, et al.* (2010). Intestinal crypt homeostasis results from neutral competition between symmetrically dividing Lgr5 stem cells. Cell *143*, 134-144.

Tumbar, T., Guasch, G., Greco, V., Blanpain, C., Lowry, W.E., Rendl, M., and Fuchs, E. (2004). Defining the epithelial stem cell niche in skin. Science *303*, 359-363.

Van Keymeulen, A., Rocha, A.S., Ousset, M., Beck, B., Bouvencourt, G., Rock, J., Sharma, N., Dekoninck, S., and Blanpain, C. (2011). Distinct stem cells contribute to mammary gland development and maintenance. Nature *479*, 189-193.

Supplemental Experimental Procedures, Table 1. List of 96 Taqman assays used for single cell RT-qPCR.

**Category**	**Gene name**	**Taqman Assay ID**
Housekeeping	*Abl1*	Mm00802029_m1
	Ppia	Mm02342429_g1
	Rab7	Mm01183732_g1
	Reep5	Mm00492230_m1
	Ubc	Mm02525934_g1
All columnar cells	*Krt8*	Mm04209403_g1
	Krt8	Mm00835759_m1
Secretory cell specific	*Bpifa1*	Mm00465064_m1
	Cbr2	Mm01246806_g1
	Cyp2f2	Mm00484087_m1
	Fut4	Mm00487448_s1
	Reg3g	Mm01181783_g1
	Scgb1a1	Mm00442046_m1
	Scgb3a2	Mm00504412_m1
Putative ciliated cell specific	*Dnali1*	Mm00613749_m1
	Fank1	Mm00482221_m1
	Foxj1	Mm01267279_m1
	Mcin	Mm01308202_m1
	Myb	Mm00501741_m1
NE cell specific	*Ascl1*	Mm04207567_g1
	Calca	Mm00801463_g1
BC-specific, or previously reported as enriched in BCs	*Arid3a*	Mm00492251_m1
	Arid3b	Mm01179426_m1
	Ascl2	Mm01268891_g1
	Barx2	Mm01176203_m1
	Bhlhb9	Mm01701581_s1
	Bnc1	Mm01324337_m1
	CEBPg	Mm01266786_m1
	EphB2	Mm01181021_m1
	EphB4	Mm01201157_m1
	Fstl3	Mm01168957_m1
	Hmga1	Mm04213075_u1
	Hmga2	Mm04183367_g1
	Hopx	Mm00558630_m1
	Icam1(CD54)	Mm00516023_m1
	Ikzf1	Mm01187881_m1
	Itga3	Mm00442910_m1
	Itga5	Mm00439820_m1
	Itga6	Mm00434375_m1
	Itgb1	Mm01253230_m1
	Itgb4	Mm01266840_m1
	Itgb6	Mm01269869_m1
	Jub	Mm00495049_m1
	Klf6	Mm00516184_m1
	Klf7	Mm00728361_s1

	Klf8	Mm01353192_m1
	Klf9	Mm00495172_m1
	Klf13	Mm00727486_s1
	Krt5	Mm01305291_g1
	Krt5	Mm00503549_m1
	Krt14	Mm00516876_m1
	Krt14	Mm00516870_mH
	Lmo1	Mm00475438_m1
	Myc	Mm00487804_m1
	Nfatc2	Mm00477776_m1
	Nfatc4	Mm00452373_g1
	Ngfr	Mm01309638_m1
	Snai2	Mm00441531_m1
	Sox2	Mm03053810_s1
	Sox4	Mm00486317_s1
	Sox7	Mm00776876_m1
	Sox15	Mm00839542_g1
	Tbx2	Mm00436915_m1
	Trp63	Mm00495791_m1
	Zfp287	Mm01334842_m1
	Zfp213	Mm01309214_m1
	Zscan20	Mm00625080_m1
	Zfp281	Mm01296016_s1
Cell cycle regulators	*Ccnd1 (CycD)*	Mm00432359_m1
	Cdkn1a (p21)	Mm04205640_g1
	Cdkn1b (p27)	Mm00438168_m1
	E2F3	Mm01138833_m1
	E2F4	Mm00514160_m1
	E2F7	Mm00618098_m1
Notch signalling	*Dll1*	Mm01279269_m1
	Dll1	Mm01279268_m1
	Dlk2	Mm01281511_g1
	Jag1	Mm00496902_m1
	Jag2	Mm01325629_m1
	Notch1	Mm00435249_m1
	Notch2	Mm00803062_m1
	Notch3	Mm01345646_m1
	Psen1	Mm01297739_m1
RTK signalling	*Egfr*	Mm00433023_m1
	Ets1	Mm01175819_m1
	Etv4	Mm01245872_m1
	Etv5	Mm00465816_m1
	Fgfr1	Mm00438930_m1
	Fgfr2	Mm01269930_m1
	Fgfr3	Mm00433294_m1
	Lrig1	Mm00456116_m1
	Spry1	Mm00839969_g1
	Spry2	Mm00442344_m1

Wnt signalling	*Dkk3*	Mm00443800_m1
	Procr (CD201)	Mm00440993_mH
	Sfrp1	Mm00489161_m1

Supplementary Note

In this Supplementary Note we present further details of the biophysical and statistical approach used to infer the lineage hierarchy and proliferation kinetics of progenitors in the trachea.

1 Stochastic Modelling

To define cell fate behaviour in the trachea we extend the biophysical modelling approach introduced in Refs. [1,2]. In this model, stem cells and their differentiating progenitor cell progeny function as equipotent cell populations in which their proliferation kinetics and fate behaviour follow defined statistical dependencies. These stochastic "rules" summarize the product of the complex gene regulatory processes that regulate cell fate behaviours. For simplicity, following Refs. [1,2], we consider a model based on intrinsic (cell-autonomous) regulation. However, in the two-dimensional geometry of the tracheal epithelium, models of fate behaviour based on external (cell extrinsic) regulation are difficult to discriminate from intrinsically regulated systems. The clonal analysis described here therefore leaves the question of the underlying regulatory dynamics unspecified.

In the following we make use of this stochastic modelling approach to infer the simplest model which is capable of describing the complex range of clonal dynamics observed in experiment. First, we analyse the dynamics in the basal layer making use of the *Tg(KRT5-CreER); Rosa26R-fGFP* line. We find that the basal population consists of two subtypes - a dividing basal stem cell (BSC) population and transient luminal precursors (BLPs). Independently, we then employ lineage

tracing data from *Scgb1a1-CreER; Rosa26R-fGFP* mice to infer the dynamics in the luminal layer. We find that secretory cells (SecCs) are lost at a surpringly high rate. Finally, we compare these findings with the full clonal data from *Tg(KRT5-CreER); Rosa26R-fGFP* mice, as well as *Krt5-CreER; Rosa26R-Confetti* and *K8-CreER; Rosa26R-fGFP* mice, and independent BrdU and EdU measurements.

1.1 Maximum likelihood estimation

The statistical approach we employ to disclose the dynamical rules that underlie fate determination in the trachea is based on the idea of *Bayesian inference* [3]. By applying this approach we seek the model that has the highest probability to describe the given experimental outcome. In other words, we aim to calculate the probability $P(\Theta|E)$ of a model (defined by dynamical rules and a set of parameters $\Theta = (\Theta_1, \Theta_2, \ldots)$) taking into account the experimental evidence E. According to *Bayes' Theorem*, this probability is proportional to the product of the probability of obtaining the experimental evidence if we assume a certain model to be true, $P(E|\Theta)$, and our *a priori* belief in a certain model, $P(\Theta)$:

$$P(\Theta|E) = \frac{P(E|\Theta)P(\Theta)}{\int_\Theta \mathrm{d}\,\Theta P(E|\Theta)P(\Theta)}\,. \tag{1}$$

The integral in the denominator ensures normalisation. We define the likelihood function $\mathcal{L}_E(\Theta) \equiv P(E|\Theta)$ by treating $P(E|\Theta)$ as a function of the models, Θ, for a given set of experimental evidence, E. The principle of maximum likelihood then states that, if we do not have any *a priori* belief in the validity of any model, i.e. the distribution $P(\Theta)$ is uniform, the model with the highest probability is given by the maximum of the likelihood function $\mathcal{L}_E(\Theta)$.

In our case, the experimental evidence is given by the frequency $f_{n_b,n_s,n_c,t}$ of observations of clones with given numbers of basal, secretory, and ciliated cells (n_b, n_s, n_c) at a given time point t after induction. Since these measurements are statistically independent, the likelihood function factorises into single observations of clones, $\mathcal{L}_{f_{n_b,n_s,n_c,t}}(\Theta) = \mathcal{N}^{-1} \prod_t \prod_{n_b,n_s,n_c} P(n_b, n_s, n_c, t|\Theta)^{f_{n_b,n_s,n_c,t}}$. The prefactor is determined by normalisation, $\mathcal{N}^{-1} = \left(\sum_{n_b,n_s,n_c,t} f_{n_b,n_s,n_c,t}\right)!/\prod_{n_b,n_s,n_c,t} f_{n_b,n_s,n_c,t}!$, where the product and sum go over all measured time points t and all cellular compositions (n_b, n_s, n_c). Hence, the likelihood function takes the form of a multinomial distribution,

$$\mathcal{L}_E(\Theta) = \frac{\left(\sum_{n_b,n_s,n_c,t} f_{n_b,n_s,n_c,t}\right)!}{\prod_{n_b,n_s,n_c,t} f_{n_b,n_s,n_c,t}!} \prod_t \prod_{n_b,n_s,n_c} P(n_b, n_s, n_c.t|\Theta)^{f_{n_b,n_s,n_c,t}}, \qquad (2)$$

How do we obtain the likelihood function $\mathcal{L}_E(\Theta)$? The dynamics of the stochastic models we consider here is described by *Master equations* of the form

$$\frac{\mathrm{d}}{\mathrm{d}t} P(n, t|\Theta) = \sum_{n'_b,n'_s,n'_c=0}^{\infty} \mathcal{W}_{n',n} P(n', t|\Theta) - \mathcal{W}_{n,n'} P(n, t|\Theta), \qquad (3)$$

determining the time evolution of the probability $P(n, t|\Theta)$ to find the clonal composition $n = (n_b, n_s, n_c)$ at time t. The quantities $\mathcal{W}_{n,n'}$ are the transition rates between states with different cellular composition and therefore encode the details of the model Θ under consideration. In the simplest cases, these equations can be solved analytically, such that $P(n_b, n_s, n_c, t|\Theta)$ is explicitly known. Generally, however, Master equations can only be solved numerically. Here, we employ stochastic simulations using *Gillespie's algorithm* [4], which allows us to compute single realisations of the stochastic dynamics. By aggregating these realisations into histograms we are able to obtain an approximation to the probability density $P(n_b, n_s, n_c, t|\Theta)$. However, in lineage tracing experiments it is not feasible to measure the number of clones that were lost before the mice are analysed.

We therefore effectively measure a conditional (persistence) probability distribution $P^p_{n>0}(n, t|\Theta)$, which is obtained by dividing P by the probability of finding a clone of size larger than zero,

$$P^p_{n>0}(n, t|\Theta) = \frac{P(n, t|\Theta)}{1 - P(0, t|\Theta)} \,. \tag{4}$$

These solutions of the Master equations give the probability to find any stochastic fate of a single clone. By substituting a particular experimental outcome into these solutions we therefore obtain the value of the likelihood function for a given model Θ and the logarithm of the likelihood function is given by

$$\log \mathcal{L}_E(\Theta) = \log \mathcal{N}^{-1} + \sum_t \sum_{n_b, n_s, n_c} N_{n_b, n_s, n_c, t} \log P^p_{n>0}(n_b, n_s, n_c, t|\Theta) \,. \tag{5}$$

In principle, to calculate $\log \mathcal{N}^{-1}$ we could made use of *Stirling's approximation*, $\log n! \approx n \log n - n$, for the numerator. However, the term $\log N^{-1}$ is independent of the model Θ. Since we are here only interested in the ratios of likelihood functions (or differences in the log likelihoods) we henceforth neglect the normalisation factor and report the unnormalised likelihood function $\tilde{\mathcal{L}}_E(\Theta) = \mathcal{N} \mathcal{L}_E(\Theta)$. To determine the maximum of the likelihood function we first searched parameter space using a simulated annealing algorithm to roughly estimate the location of the most likely parameters. Once the location of the maximum likelihood parameters had been narrowed down, we rasterised parameter space surrounding these optimal parameters to precisely determine the best fit parameters and to calculate credible intervals.

1.2 Uncertainty and credibility intervals

The maximum likelihood estimation is subject to various sources of uncertainty, most importantly finite sample sizes of experimental and numerical data. In addition, there is a significant variability

between mice. To account for this uncertainty we give parameter estimates with margins denoting the uncertainty, which is associated with these values. Specifically, in the spirit of a likelihood ratio test, we consider all parameter values as acceptable whose likelihood is above a given threshold, α, such that the resulting credible region (CR) is

$$\text{CR} = \left\{ \Theta \;\middle|\; \frac{\mathcal{L}_E(\Theta)}{\mathcal{L}_E(\Theta^*)} > \alpha \right\} . \tag{6}$$

It has been shown that this approach is most powerful in classifying sets of credible parameters [3].

We here choose $\alpha = 0.05$, such that the credible region includes parameter values whose likelihood is larger than 5% of the maximum likelihood value. The dynamics of cells in the trachea is defined by several parameters. Hence, for a specific value of one of these parameters, Θ_i, we give credible intervals in the form $\Theta_i^* \pm (\max_{\text{CR}}\{\Theta_i\} - \Theta_i^*, \Theta_i^* - \min_{\text{CR}}\{\Theta_i\})$.

1.3 Regional variability

Clones were regionally specified according to whether they were located over cartilage rings or over the dorsal longitudinal muscle. To test if the clonal dynamics in these two regions differs significantly, we calculated the probability that for each time point and cell type the resulting probability distributions are equal. To calculate this probability we employed a Mann-Witney U-test. The resulting p-values are given in Table 1. From 24 different statistical tests for all time points and cell types, only three indicated statistically significant differences (p-value < 0.05). Two of these three involve mice analysed at 1 year after induction. Inspection of the average clone

Time [days]	3	21	42	85	188	275	366	517
Basal	-	0.22	0.02	0.40	0.42	0.28	0.002	0.22
Secretory	-	0.44	0.45	0.15	0.07	0.18	0.60	0.66
Ciliated	-	-	0.20	0.75	0.92	0.16	0.01	0.33

Table 1: P-values for comparing clone sizes observed in regions overlying cartilage rings and clones overlying dorsal longitudinal muscle band.

sizes for each time point (Fig. 1g) reveals that the 1 year time point is likely not representative for the overall clonal dynamics. Since most deviations between the different regions in the trachea can therefore be explained by statistical variation it is reasonable to assume that there are no significant differences in the mechanisms of fate choice. In the following we therefore pool the data for both regions.

1.4 Clonal merging

To analyse the clonal fate data it is important to take into account possible ambiguity due to clonal mergers. In the following we estimate the frequency of mergers in regions overlying the dorsal longitudinal muscle band in which clones have the highest induction frequency and are therefore particularly prone to merging events. For muscle regions we find roughly 40 patches in an area of 630 µm x 630 µm, which translates to a typical distance between clones of about $a \approx 100$ µm. With this, the distance r between neighbouring clones follows a Rayleigh distribution, $G(r) = 2r/a^2 \exp\left[-(r/a)^2\right]$. The probability of merging of two clones of size R is then given by the probability that a randomly chosen clone overlaps with its nearest neighbour. This

probability is just the cumulative distribution, i.e. the probability that their distance is smaller than the sum of their radii, $P_R^{\text{merge}} = \int_0^R G(r)\,\mathrm{d}r = 1 - \exp\left[(R/a)^2\right]$. Since a single cell covers an area of approximately $s = 120$ µm^2 we can re-express the probability of merging as $1 - \exp(ns/a^2)$. For an average clone size of 10 cells this yields a probability of merging of roughly 10%, indicating that clonal mergers are rare but not negligible. However, this simple estimation does not take into account the fact that large clones occur at a lower frequency than small clones. In the absence of mergers we expect the clone sizes to be exponentially distributed. Mergers have a larger clone size than expected from an exponential distribution and the clone size above which mergers become relevant is characterized by the deviation of the empirical clone size distribution from the exponential form. Following this approach we therefore neglected all clones that had more than 19 cells of a given type.

For the *Scgb1a1-CreER* experiment, the clonal density was considerably lower, with on average 37 clones per mm^2 in muscle areas and 29 clones per mm^2 in cartilage areas. While the exact form of the distribution of clone sizes is hard to read from the data, we again approximated this distribution by an exponential with an average clone size of $\langle n \rangle \approx 1.5$, roughly independent of time. Since luminal cells have a similar area in the xy plane as basal cells (120µm^2) we find that the frequency of mergers is roughly 2% in both areas. For the *Scgb1a1-CreER* dataset we may therefore neglect the influence of merging events.

1.5 Inference of the best fitting model

We are now in a position to employ statistical inference in order to estimate the model, which describes the experimental data with the highest likelihood. Since the trachea consists of several cell types, each potentially exhibiting a variety of different behaviors, the parameter space we have to search for the best model is high-dimensional, which renders a simultaneous estimation of the maximum likelihood parameters impossible. To make progress we therefore employ different lineage tracing experiments to independently infer the dynamics in the basal and the luminal layer. Moreover, the range of possible models is greatly constrained by the requirement that the lung tissue is homeostatic in adult mice.

1.5.1 Basal layer

We start our analysis with the basal layer. In the *Tg(KRT5-CreER); Rosa26R-fGFP* line, basal cells are genetically labelled. This label is, upon cell division, inherited by all progeny of these cells. If we neglect the loss of clones we can interpret the average number of basal cells in analysed clones as the average number of basal cells in *all* clones. In homeostasis, we would expect that once a basal cell is labelled the number of basal cells, averaged over all clones, remains constant at a value of unity. What we find, however, is an increase of the average number of basal cells to a value of two, cf. Fig. 1g. This behaviour is not consistent with a single basal population in a homeostatic tissue. Rather, this increase in the average number of basal cells strongly suggests that the basal population consists of at least two cell types of which only one is labeled.

To describe the dynamics in the basal layer we therefore consider two cell populations: basal stem cells (BSCs), denoted by A, and basal luminal precursors (BLPs), denoted by B. The existence of two basal populations will later be independently supported by our statistical analysis. Further, as noted in the main text, we unambiguously identified this second population via the genetic marker K8. In adult tissue, self-renewal of stem cells must be tightly balanced with differentiation to maintain homeostasis. In principle, there are two possible ways in which this balance can be achieved: on the level of single cells by asymmetric divisions, or on the population level, if symmetric divisions and differentiation happen at exactly the same rate. Taking into account both modes of division we are left with the following possible dynamical rules for basal stem cells:

$$A \to \quad AA \quad \text{with a rate } \alpha_1, \tag{7}$$

$$A \to \quad AB \quad \text{with a rate } \alpha_2, \tag{8}$$

$$A \to \quad BB \quad \text{with a rate } \alpha_3, \tag{9}$$

where homeostasis dictates that $\alpha_1 = \alpha_3$.

What are the dynamical rules governing the behaviour of the second basal population? As discussed below, we can rule out asymmetric divisions of the second basal population from EdU experiments. Also, to maintain homeostasis, basal progenitors must differentiate at the same rate that they are created and leave the basal layer. For now, we leave the details of this differentiation process to a later discussion. We note that differentiation via asymmetric division does not change the number of basal progenitors and that any direct differentiation processes can by effectively

summarised by loss of basal progenitors:

$$B \to \emptyset \text{ with a rate } \beta. \tag{10}$$

If N_b is the total number of basal cells in a sufficiently large frame of tissue, the loss of basal progenitors per unit time and per basal cell is given by the rate of loss of single basal progenitors, β, times the fraction of basal progenitors in the tissue, b, where the concentration b is defined such that $N_b \cdot b$ is the total number of basal progenitors. This overall loss rate of progenitors, βb, must exactly match the overall production rate of progenitors through differentiation of basal stem cells, $a(2\alpha_1 + \alpha_2)$. In homeostasis,we therefore require

$$\beta = \frac{a}{b}\left[2\alpha_1 + \alpha_2\right], \tag{11}$$

where a/b denotes the relative abundances of basal stem cells and basal progenitors.

For the basal layer we are left with three parameters to be determined by statistical inference: α_1, α_2, and a/b. Performing the maximum likelihood estimation we find that the best fitting parameters correspond to a log likelihood of $\log \mathcal{L}_E(\Theta^*) = -2077$, with rates

$$\begin{aligned}
\alpha_1 &= 0.0029 \pm (0.0006, 0.0008)\,\mathrm{d}^{-1}, \\
\alpha_2 &= 0.085 \pm (0.05, 0.025)\,\mathrm{d}^{-1}, \\
\beta &= 0.095 \pm (0.062, 0.037)\,\mathrm{d}^{-1}, \\
a/b &= 1.05 \pm (0.25, 0.25)\,\mathrm{d}^{-1}.
\end{aligned} \tag{12}$$

The uncertainty for α_2 is much larger than for α_1 since, after a short time, the clonal dynamics approaches a scaling regime, where the distribution of the size of persistent basal clones takes a

simple scaling form [2],

$$\lim_{t \gg 1/\alpha_1} P_{n>0}^{\mathrm{pers}} = (\alpha_1 t)^{-1} f\left[(\alpha_1 t)^{-1} n)\right] . \tag{13}$$

This is independent of the parameter α_2 which must therefore be determined from the early time points alone.

In summary, we find that basal stem cells divide on average every $11 \pm (4,4)$ d. Of these divisions, the vast majority, $93.6 \pm (2.7, 2.3)\%$, result in asymmetric fate outcome. A basal progenitor differentiates on average every $11 \pm (7,4)$ d.

Having determined the optimal parameters defining a model for the basal layer involving two basal populations we now ask whether the basal dynamics can also be described by a model involving a homogeneous basal population. To this end we consider a single population, A, undergoing symmetric and asymmetric divisions: $A \to AA$ and $A \to A\emptyset$, where the second process summarises all loss of basal cells through differentiation or cell death. In homeostasis, both processes must be precisely balanced and happen at the same rate, α. Again, asymmetric divisions do not change the number of basal cells and may therefore be neglected. Statistical inference reveals that, given a homogeneous basal population, the maximum likelihood parameter is $\alpha = 0.016 \pm (0.0007, 0.0018)\,\mathrm{d}^{-1}$ with a corresponding log likelihood of $\log \tilde{\mathcal{L}}_E(\Theta^*) = -2325$. Comparing this result to the maximum likelihood estimate obtained for the heterogeneous basal population (-2077), the difference corresponds to a Bayes factor of $K = 5 \cdot 10^{107}$ or $2 \ln K = 496$, which is highly decisive in favour of a heterogeneous basal population.

While our simple model provides an excellent prediction of the clonal data, we cannot rule out

the possibility of a more complex dynamics in the basal layer. Indeed, many more complicated models yield clone size distributions which quickly relax to the same scaling form that arises from our simple model [2]. However, we can make progress by making use of the fact that the overall rate of cell divisions is constrained by BrdU measurements to roughly 0.09 d^{-1}. By keeping the overall rate of cell divisions fixed we first asked whether reversibility, i.e. the "de-differentiation" of BLPs to BSCs ($B \to A$) could capture the clonal data equally well. Our stochastic simulations suggested that the rate of such reversibility processes, would they exist, must be lower than 0.001 d^{-1}. It therefore seems unlikely that BLPs de-differentiate to BSCs, at least under conditions of normal homeostasis.

We then asked whether the clonal data is compatible with proliferating BLPs, again fixing the overall frequency of cell divisions in the basal layer. To this end, we extended our model by a process involving symmetric divisions of BLPs, $B \to BB$, while adjusting the rate of loss of BLPs such that homeostasis is maintained. From the clonal data we cannot rule out symmetric divisions of BLPs. However, their rate is constrained to less than 0.045 d^{-1}, which is less than 50% of the total number of cell divisions in the basal layer. In particular, this suggests that the BLPs are not a traditional transit amplifying cell population. This view is supported by H2B-GFP measurements, as discussed below.

1.5.2 Luminal layer

The relatively large degree of uncertainty in the rate of differentiation from the basal layer, β, makes straightforwardly extending the basal dynamics to the luminal layer impossible. We therefore performed independent experiments making use of a *Scgb1a1* promoter, which labels secretory cells and their progeny. With SeCs induced sporadically at a constant rate, this translates to a mathematical theory of branching processes with immigration [5], which predicts that the observed clone size distributions should be stationary. Indeed from the clonal data, we find that the probability distributions of cell numbers in the luminal layer are essentially time-independent.

We first note that there are no clones involving basal cells and we can therefore exclude the possibility of de-differentiation of secretory cells in homeostasis. The probability distribution $\tilde{P}(n_s, n_c, t)$ of finding n_s secretory cells and n_c ciliated cells at a time t after induction can then be obtained by integrating over the time course,

$$\tilde{P}(n_c, n_s, t) = \int_0^t \mathrm{d}\tau P(n_s, n_c, t-\tau) Q(t-\tau)\,, \tag{14}$$

where $P(n_s, n_c, t-\tau)$ denotes the distribution of clones at a time $t-\tau$ after induction and $Q(t-\tau)$ is the probability that a clone is induced at a time $t-\tau$. We here set $Q(t-\tau) = 1/t$, i.e. clones are continuously induced with the same probability at each time point.

To begin we focus on secretory cells, S, alone. The range of possible dynamics is constrained by a remarkably low loss rate at the end of the lineage: a ciliated cell is, on average, lost once every 6 months, translating to a loss rate of $\gamma = 0.0038\,\mathrm{d}^{-1}$, which is by a factor of 20 smaller

than the rate of cell divisions found for the basal layer. This already restricts the rate of potential divisions of ciliated cells, as well as the total rates differentiation to ciliated cells to less then $0.0038\,\mathrm{d}^{-1}$. Hence, within a statistical analysis these rates can not be distinguished from 0 and we may therefore neglect them in the ensuing analysis. Since we know from experiments that ciliated cells do not divide we end up with the following dynamical processes for the luminal layer:

$$S \to \quad SS \quad \text{with a rate } \sigma_1, \tag{15}$$

$$S \to \quad SC \quad \text{with a rate } \sigma_2, \tag{16}$$

$$S \to \quad CC \quad \text{with a rate } \sigma_3, \tag{17}$$

$$S \to \quad \emptyset \quad \text{with a rate } \sigma_4, \tag{18}$$

$$C \to \quad \emptyset \quad \text{with a rate } \gamma. \tag{19}$$

Fortunately, homeostasis again constrains the number of free parameters. In order for the population of secretory cells to remain constant, their total production rate, $s\sigma_1 + b\beta$, must equal their total loss rate, $s(\sigma_3 + \sigma_4)$, i.e.

$$\sigma_3 = \frac{b}{s}\beta + \sigma_1 - \sigma_4. \tag{20}$$

The ratio of basal progenitors to secretory cells can not be directly inferred from cell counting as the morphology of basal stem cells and basal progenitors is indistinguishable. However, with the ratio of all basal stem cells and basal progenitors, $a/b \approx 1.05$ and the ratio of basal cells to secretory cells, $(a+b)/s \approx 1$, we can rewrite b/s in terms of known quantities, namely the ratio of basal stem cells to progenitors and the ratio of basal cells to secretory cells,

$$\frac{b}{s} = \frac{b}{a+b}\frac{a+b}{s} = \frac{a+b}{s}\frac{1}{a/b+1}\,. \tag{21}$$

Since σ_3 should be greater or equal to zero, the condition of homeostasis not only fixes the value of σ_3 but also constrains the loss rate of secretory cells,

$$\sigma_4 \leq \frac{b}{s}\beta + \sigma_1. \tag{22}$$

Stationarity of the ciliated cell population requires that

$$\sigma_2 = \frac{c}{s}\gamma - 2\sigma_3, \tag{23}$$

and making use of Eq. (20), we obtain

$$\sigma_2 = \frac{c}{s}\gamma - 2\left[\frac{b}{s}\beta + \sigma_1 - \sigma_4\right] \tag{24}$$

Since σ_2 must be greater than zero we obtain another constraint on the value of the loss of secretory cells,

$$\sigma_4 \geq \frac{b}{s}\beta + \sigma_1 - \frac{\gamma c}{2s} \tag{25}$$

Together with Eq. (22) this constrains possible values of σ_4 to an interval of length $\gamma c/(2s)$. Indeed, since it has been found that 50% of ciliated cells are lost within six months, i.e. $\gamma \approx 0.0038\,\mathrm{d}^{-1}$, the width of this interval is much smaller than the actual value of σ_4 and its uncertainty, which is mostly determined by the uncertainty in β. Since according to EdU measurements there is no indication for symmetric, differentiating divisions of secretory cells we fixed the value of σ_4 such that σ_3 vanishes, namely to the upper end of this interval,

$$\sigma_4 \approx \frac{b}{s}\beta + \sigma_1 \tag{26}$$

We finally obtain

$$
\begin{aligned}
\sigma_2 &\approx \frac{c}{s}\gamma, \\
\sigma_3 &\approx 0, \\
\sigma_4 &\approx \frac{a+b}{s}\frac{1}{a/b+1}\beta + \sigma_1,
\end{aligned}
\tag{27}
$$

with the only remaining parameter to be determined by statistical inference being the rate of symmetric divisions of secretory cells, σ_1. Already at this point we can therefore say that the rate of asymmetric divisions and the rate of symmetric differentiation is small, while the loss of secretory cells compensates for potential symmetric divisions and the differentiation of basal cells.

To determine the rate of symmetric divisions of secretory cells, σ_1, we performed stochastic simulations to obtain it's maximum likelihood estimate. We find that the value of maximum likelihood is $\sigma_1 = 0.037 \pm (0.010, 0.008)$. Hence, a secretory cell divides on average every $25 \pm (7, 5)$ d. $93 \pm (2, 2)\%$ of these divisions are symmetric resulting in two secretory cells, while $8.6 \pm (2.9, 1.7)\%$ lead to asymmetric fate outcome. The proliferative capacity of secretory cells is compensated by a large loss rate: a secretory cell is lost on average every $14 \pm (2, 2)$ d.

1.5.3 The full lineage

Having defined the dynamics in the basal layer and the luminal layer separately we now establish how these two layers are connected. Since BrdU measurements reveal that our model of the basal layer encompassed the majority if not all of the possible cell divisions, further proliferation of basal progenitors seems unlikely. It also seems unlikely that basal cells are lost at a significant rate. We

therefore propose that basal progenitors differentiate to secretory cells at a rate $\beta = 0.095\,\mathrm{d}^{-1}$. Indeed, by assuming this simplest possible link between the basal layer and the luminal layer were are able to obtain an excellent fit to the full clonal data from the *Tg(KRT5-CreER); Rosa26R-fGFP* mice (Fig. 2e and S4).

Interestingly, close inspection of the fits for the secretory cells reveals that we slightly, but consistently, underestimate the frequency of clones with a single secretory cell. Note that the number of secretory cells in a given clone was defined as the number of cells that were not identified as basal or ciliated cells. We therefore speculate that the abundance of singlets of secretory cells can be explained by the the existence of another cell type in the lineage, which are likely neuroendocrine or brush cells. The existence of an additional (but infrequent) persistent cell type also explains why clonal loss is overall rare (cf. Fig. 1g), while our model predicts a relatively significant loss.

1.6 Deviation from homeostasis

Measuring the cellular composition in mice of different ages we find that the ratio of ciliated cells to secretory cells, c/s, changes from 0.83 in young mice to about 0.5 in mice 1 year year after injection. We now ask what effect such a deviation from homeostasis has on the values of the model parameters. In the framework of our model, such a deviation is caused by an imbalance in the rates of symmetric divisions, leading to an exponential departure from the homeostatic cellular composition. Since this departure is exponential, we expect that its influence on the model parameters is only logarithmic, i.e. very weak. Importantly, according to our analysis, the value of c/s only affects the dynamics in the luminal layer. We therefore performed the maximum like-

lihood estimation for the maximum and the minimum value of c/s. For $c/s = 0.83$ we obtained $\sigma_1 = 0.036 \pm (0.011, 0.008)$, while for $c/s = 0.5$ we found $\sigma_1 = 0.040 \pm (0.013, 0.008)$. Both results are in good agreement with the maximum likelihood value of $0.037 \pm (0.0105, 0.008)$ for $c/s = 0.75$, which we chose to fit our model. Hence, the change in cellular compositions does not significantly affect the estimation of parameters.

2 Consistency checks

While our model faithfully predicts the full clone size distributions and their cellular composition over time, it is important to check the validity of our results by independent experiments.

2.1 Krt5- CreER; Rosa26R-Confetti and K8-CreER; Rosa26R-fGFP experiments

To test whether our model could predict the outcome of independent lineage tracing experiments we made use of *Krt5- CreER; Rosa26R-Confetti* and *Krt8-CreER; Rosa26R-fGFP* experiments mice. For the *Krt5- CreER; Rosa26R-Confetti* line we found excellent agreement between the model predictions and the observed distributions of clone sizes (Fig. 3d). In the*Krt8-CreER; Rosa26R-fGFP* line both, SecCs and CCs were induced at a rather high frequency. As a result, labelled cells at 4 d post induction occurred not only in singlets, but also in doublets and triplets, even for non-dividing CCs. To take account for these mergers we drew the initial conditions of our simulations randomly from the clone size distribution given by the 4d post induction time point. We also observed a small degree of background labelling, particularly at late time points, which is supported by the fact that clone size distributions change little over time [5]. Therefore, not too long

after induction the clone size distribution should be dominated by clones from the initial labelling. Indeed, our model does a good job at predicting these early clones. By contrast, at late time points clones dominate that had been induced by background labelling at earlier time points. Assuming background induction at the same relative frequency between SecCs and CCs as initial labelling our model excellently predicts the long time clonal dynamics (Fig. 3f).

2.2 BrdU experiments

We can challenge these results using independent nucleotide incorporation assays. From our measured steady-state BrdU incorporation rates (Supplemental S1) we can infer the fraction of cells in S-phase. The measured fraction $f \approx 1.3\%$ relates to the cell cycle time of dividing cells, T_c via the time cells spend in S-phase, which we take to be around 7 hours,

$$T_c = \frac{T_S}{2f} \approx 11\,\text{days}\,. \tag{28}$$

We divided by 2 as, according to the modelling results, only about 50% of the basal cells undergo divisions. In the stochastic model the average cell cycle time is $(2\alpha_1 + \alpha_2)^{-1} \approx 11\,\text{d}$, in excellent agreement with the BrdU measurements.

2.3 EdU experiments

Having confirmed the overall rate of cell divisions in the basal layer we now ask whether our model is also able to predict the distribution of different division modes. To identify the types of cell divisions made in the wildtype trachea at steady-state we combined EdU incorporation with wholemount immunostaining to visualize S-phase cells (2 hours post-EdU, Fig. 2h and i, left) and

their immediate progeny (24 hours post-EdU, Fig. 2i, right). Based on our model we can calculate the predicted ratios of labelled single cells 2h after EdU incorporation. To this end we note that the fraction of basal cells among all cells in S-phase is given by the fraction of dividing basal cells in the whole population divided by the fraction of dividing cells in the tissue. In the whole population the fraction of dividing basal cells is equal to fraction of basal cells, which are capable to divide, r_b, multiplied by the probability that a basal cells is in S-phase, $T_s\lambda_b$. Here, T_s is the time a cell spends in S-phase (typically 6-8h) and $\lambda_b = 2\alpha_1 + \alpha_2$ is the total division rate of basal cells. Since, according to the model, secretory cells are the only other dividing cell type, the total fraction of dividing cells in the tissue is $T_s(r_b\lambda_b + r_s\lambda_s)$, where r_b denotes the fraction of secretory cells and $\lambda_s = \sigma_1 + \sigma_2$ is their total division rate. Therefore, we predict that after 2h of all labelled singlets $r_b\lambda_b/(r_b\lambda_b + r_s\lambda_s)$ are basal cells, $r_s\lambda_s/(r_b\lambda_b + r_s\lambda_s)$ are secretory cells and none are ciliated cells. Indeed, we find an excellent agreement with the EdU measurements (Fig. 2i).

Since all average division times are on the order of weeks we expect that 24h post EdU the cells have undergone exactly one division. The fact that at this time point we observe a considerable fraction of secretory cell singlets strongly suggests that EdU is toxic to secretory cells. We therefore focus our analysis on the relative fractions of different types of doublets in the basal layer and the luminal layer. According to our model, dividing cells in the basal layer will always produce two daughter cells in the basal layer, but no combinations of basal cells and secretory cells. We find that this prediction is in excellent agreement with the relative fractions of these division modes obtained from the EdU experiments (Fig. 2i). We now turn to the luminal layer. Here, our model predicts that 93% of divisions of secretory cells generate two secretory daughter cells, 7% produce

a secretory cell and a ciliated cell and none leads to a pair of ciliated cells. Again, this is in almost perfect agreement with the ratios we obtained from the EdU experiments.

With these results we can now ask whether there is a possibility for CC production through BLPs. To answer this we note that in homeostasis the loss of ciliated cells must be balanced by production form different sources, $\gamma c = \sigma_2 s + \epsilon b$. Here, ϵ denotes the production rate of CCs by BLPs. We now consider the minimum percentage of asymmetric divisions of SCs consistent with the EdU experiment, which we define as the lower bound of the 95% confidence intervals (6.6%). With the overall rate of SC divisions from our model we find that the minimum rate of CC production by SCs allowed by the EdU experiments is $\sigma_{2,\mathrm{min}} \approx 0.0026$ 1/d. With this, the maximum rate of CC production by basal cells is

$$\epsilon_{\mathrm{max}} = d/b\,\gamma - c/b\,\sigma_{2,\mathrm{min}} = 4.2 \cdot 10^{-4}\,\mathrm{d}\,. \tag{29}$$

This means that by the EdU experiments and the overall rate of SC divisions from the model the rate of CC production by BLPs is constrained to less than once per 6 years, corresponding to less than 1% of the total CC production.

In summary, the results from the EdU experiments excellently agree with our model predictions. This strongly suggests that our modelling framework contains the most important modes of cell divisions. Together with the BrdU experiments, which fix the overall rate of cell division in the basal layer, these results reassure us that there are no relevant further division modes, such as divisions of basal progenitors, or divisions of basal stem cells which contribute to the luminal layer.

2.4 H2B-GFP experiments

Using *Krt8-H2B-GFB* marking BLPs we then asked whether there was evidence for proliferation of BLPs. To this end we counted the frequencies of GFP positive cells at several time points after induction. Our model predicts that K8 expressing basal cells do not divide and the rate of loss of labelled cells therefore depends linearly on their concentration, $p(t)$ and the rate of loss of BLPs from the basal layer, $\dot{p}(t) = -\beta p(t)$. Therefore, the percentage of labelled cells should decrease exponentially with a rate corresponding to the rate of loss of cells from the basal layer, β, viz. $p(t) \propto \exp(-\beta t)$. Indeed, fitting an exponential to the H2B-GFP time course gives an average lifetime of BLPs of $16\,\mathrm{d}$, which is in good agreement with the predicted lifetime of $11\,\mathrm{d}$. We therefore believe that BLPs do not divide significantly in homeostasis.

References

1. Klein, A. M. & Simons, B. D. Universal patterns of stem cell fate in cycling adult tissues. *Development* **138**, 3103–11 (2011).

2. Klein, A., Doupé, D., Jones, P. & Simons, B. Kinetics of cell division in epidermal maintenance. *Phys. Rev. E* **76**, 021910 (2007).

3. Box, G. E. P. & Tiao, G. C. *Bayesian inference in statistical analysis*, vol. 40 (John Wiley & Sons, 2011).

4. Gillespie, D. T. Exact stochastic simulation of coupled chemical reactions. *J. Phys. Chem.* **81**, 2340–2361 (1977).

5. Bailey, N. T. J. *The Elements of Stochastic Processes with Applications to the Natural Sciences* (John Wiley & Sons, 1990).
